# Genome-Wide Identification of the *R2R3-MYB* Gene Family in Mango and Multi-Omics Analyses Reveal Genes Involved in Anthocyanin Biosynthesis in Fruit Peels of Different Colors

**DOI:** 10.3390/plants15213427

**Published:** 2026-11-04

**Authors:** Jiangtao Gai, Yanping Zheng, Kunkun Zhao, Xiuxu Ye, Jingxiao Fu, Yingbo Zhang, Huarui Chen, Zhiguo Dang

**Affiliations:** Tropical Crops Genetic Resources Institute, Chinese Academy of Tropical Agricultural Sciences, Key Laboratory of Tropical Crops Germplasm Resources Genetic Improvement and Innovation of Hainan Province, Ministry of Agriculture and Rural Affairs, Key Laboratory of Crop Gene Resources and Germplasm Enhancement in Southern China, Haikou 571101, China; gjt006616@catas.cn (J.G.); 13682060772@163.com (Y.Z.); kkzhao@catas.cn (K.Z.); yexx0405@catas.cn (X.Y.); fjx0909@catas.cn (J.F.); zhangyingbo1984@163.com (Y.Z.)

**Keywords:** *Mangifera indica*, fruit peel color, anthocyanin, R2R3-MYB, *MiMYB107*

## Abstract

Mango (*Mangifera indica* L.) is an economically important tropical fruit, and anthocyanins are key determinants of peel color. Although R2R3-MYB transcription factors (TFs) are well-established regulators of anthocyanin biosynthesis in various plant species, their roles in mango remain largely unexplored. In this study, 370 *MiMYB* genes were identified in the *M. indica* genome, including 194 *MiR2R3-MYB* genes that were unevenly distributed across 20 chromosomes. Phylogenetic analysis classified *MiR2R3-MYB* genes into 39 subfamilies, while gene structure analysis revealed their conserved organization. Tandem and segmental duplication were the primary drivers of gene family expansion, whereas the ratio of nonsynonymous to synonymous substitutions (*Ka/Ks)* suggested that these genes had predominantly undergone purifying selection. Transcriptomic analysis of red, yellow, and green mango peels identified differentially expressed *MiR2R3-MYB* genes and anthocyanin structural genes. Additionally, 143 potential MYB-binding sites were predicted in the promoters of anthocyanin structural genes. Integrated transcriptomic and metabolomic analyses revealed significant correlations between 45 differentially expressed *MiR2R3-MYB* genes and 36 differentially accumulated anthocyanin metabolites. Based on the number of significantly correlated anthocyanin metabolites, eight candidate genes were selected for validation by reverse transcription quantitative real-time PCR (RT-qPCR). Notably, the *MiMYB107* candidate gene showed positive correlations with the largest number of anthocyanin components. Subcellular localization analysis of the MiMYB107-eGFP fusion protein indicated predominant nuclear localization, and Western blotting confirmed MiMYB107 protein expression. This study provides valuable insights into the molecular mechanisms underlying anthocyanin accumulation in mango fruit peels.

## 1. Introduction

Mango (*Mangifera indica* L.), a prominent member of the Anacardiaceae family, is one of the most economically important tropical fruit crops. Fruit color is a key quality trait that strongly influences the market value and consumer acceptance of mango. Mango peel color is primarily associated with anthocyanin accumulation. In many fruit species, including mangoes [1,2], bananas [3], pears [4], and blood oranges [5], peel color development is closely associated with increased anthocyanin biosynthesis and accumulation. Consequently, elucidating the mechanisms underlying anthocyanin biosynthesis and regulation is essential for understanding the molecular basis of mango peel coloration and improving fruit quality.

In plants, anthocyanin biosynthesis is regulated by the coordinated expression of structural genes and a complex network of transcription factors (TFs), with MYB proteins playing a particularly important role in this process [6]. The MYB DNA-binding domain comprises one to four highly conserved repeats, each approximately 52 amino acids long. MYB TFs are generally classified into four groups based on the number of repeats: 1R-MYB, R2R3-MYB, 3R-MYB, and 4R-MYB. Among these, R2R3-MYB proteins constitute the largest subfamily and participate in diverse biological processes, such as secondary metabolism and stress responses. In R2R3-MYB proteins, the highly conserved DNA-binding domain is typically located at the N-terminus. In contrast, the C-terminal region is more variable among species and often contains motifs associated with transcriptional activation or repression [7].

Extensive research has demonstrated that MYB TFs play an essential role in regulating anthocyanin biosynthesis. In ‘Nanhong’ pear, *PuMYB93* positively regulates anthocyanin biosynthesis and promotes red peel pigmentation [8]. Similarly, *PpMYB5* promotes anthocyanin biosynthesis in ‘Red zaosu’ pear by synergistically activating *PpCHS* expression [4]. *PpMYB114* also enhances anthocyanin biosynthesis in pear calli by inducing *PpMYB10* expression, whereas *PpMYB10* silencing abolishes this effect, indicating that *PpMYB10* is essential for *PpMYB114*-mediated anthocyanin biosynthesis [9]. In banana, a five-member R2R3-MYB network comprising *MusaUP1*, *MusaUP2*, *MbaMA2*, *MusaMA4*, and *MusaMA8* differentially regulates anthocyanin biosynthesis in bracts [3]. *DoMYB75* also directly targets and activates the *DoANS* promoter, promoting anthocyanin accumulation in *Dendrobium officinale* [10]. MYB TFs can also repress anthocyanin biosynthesis: in apple, *MdMYB73* competes with *MdMYB1* for binding to the *MdUFGT* promoter, reducing *MdUFGT* expression [11]. In grape, *VvMYB3* competes with *VvMYBA1* for interaction and mediates histone deacetylation at the *VvUFGT* promoter, limiting anthocyanin accumulation [12]. Transient expression of *Ficus carica FcMYB57* in strawberry has likewise provided evidence that it negatively regulates anthocyanin biosynthesis [13].

Previous studies have implicated several MYB transcription factors in mango fruit pigmentation. *MiMYB10* has been reported to regulate carotenoid biosynthesis and chlorophyll degradation [14], whereas *MiMYB33* and *MiMYB1* are associated with flavonoid and anthocyanin accumulation in mango peel, respectively [1,15]. However, the genome-wide identification and evolutionary characteristics of the *MiR2R3-MYB* family, their associations with anthocyanin metabolites across peel color phenotypes, and their potential regulation of anthocyanin structural genes remain poorly characterized. To address these gaps, in this study, we integrate genomic, transcriptomic, metabolomic, and experimental analyses to investigate *R2R3-MYB* genes and their potential roles in mango peel pigmentation. Specifically, the integration of transcriptomic and metabolomic data facilitates the association of differentially expressed *R2R3-MYB* genes with specific anthocyanin compounds, thereby aiding in the identification of candidate genes involved in peel coloration.

In this study, we used the ‘Alphonso’ mango genome as a reference to identify 194 *MiR2R3-MYB* genes and systematically characterized their physicochemical properties, gene structures, phylogenetic relationships, duplication events, orthology, and synteny. Subcellular localization predictions indicated that all MiR2R3-MYB proteins localize to the nucleus, while physicochemical analyses suggested that they are generally hydrophilic and unstable. Phylogenetic analysis classified the genes into 39 subfamilies. Evolutionary analyses indicated that tandem and segmental duplications contributed substantially to the expansion of the *MiR2R3-MYB* family. In addition, all ratios of nonsynonymous to synonymous substitutions (*Ka/Ks*) were below 0.5, suggesting that these genes evolved primarily under purifying selection. Using transcriptome data, we analyzed differentially expressed genes (DEGs), including *MiR2R3-MYB* transcription factor genes and structural genes involved in anthocyanin biosynthesis. We also predicted potential MYB-binding sites in the promoters of anthocyanin structural genes. Integrated transcriptomic and metabolomic analyses of red, yellow, and green mango peels identified 45 *MiR2R3-MYB* DEGs significantly correlated with 36 differentially accumulated anthocyanin metabolites. RT-qPCR analysis of eight candidate genes showed expression trends consistent with the transcriptomic data. Among these genes, *MiMYB107* was prioritized as a candidate gene associated with anthocyanin accumulation because it was significantly and positively correlated with more anthocyanin metabolites than any other gene. Its predominant nuclear localization is consistent with its potential transcription factor role, and Western blotting confirmed expression of the MiMYB107–eGFP fusion protein. Collectively, this study identifies candidate *R2R3-MYB* genes potentially involved in anthocyanin accumulation in mango and provides a foundation for their functional characterization.

## 2. Results

### 2.1. Genome-Wide Identification and Chromosomal Distribution of R2R3-MYB Genes in M. indica

Genome-wide identification in *M. indica* revealed 370 *MiMYB* genes, comprising 171 1R-MYBs, 194 R2R3-MYBs, four 3R-MYBs, and one 4R-MYB gene (Table S1). The *R2R3-MYB* genes were sequentially designated *MiMYB1*–*MiMYB194* according to their physical positions on the corresponding chromosomes. Detailed information on the physicochemical properties of the encoded proteins is presented in Table S2. The identified protein lengths ranged from 145 amino acids for MiMYB157 to 552 amino acids for MiMYB174. The molecular weights of the R2R3-MYB proteins ranged from 17,073.61 Da (MiMYB157) to 61,104.03 Da (MiMYB174), while their theoretical isoelectric points ranged from 4.40 (MiMYB123) to 10.14 (MiMYB189). Most R2R3-MYB proteins were predicted to be unstable (instability index > 40) and hydrophilic, as indicated by grand average of hydropathy (GRAVY) values below zero. Their aliphatic index values ranged from 50.97 to 90.27, suggesting relatively high predicted thermostability.

**Figure 1:**
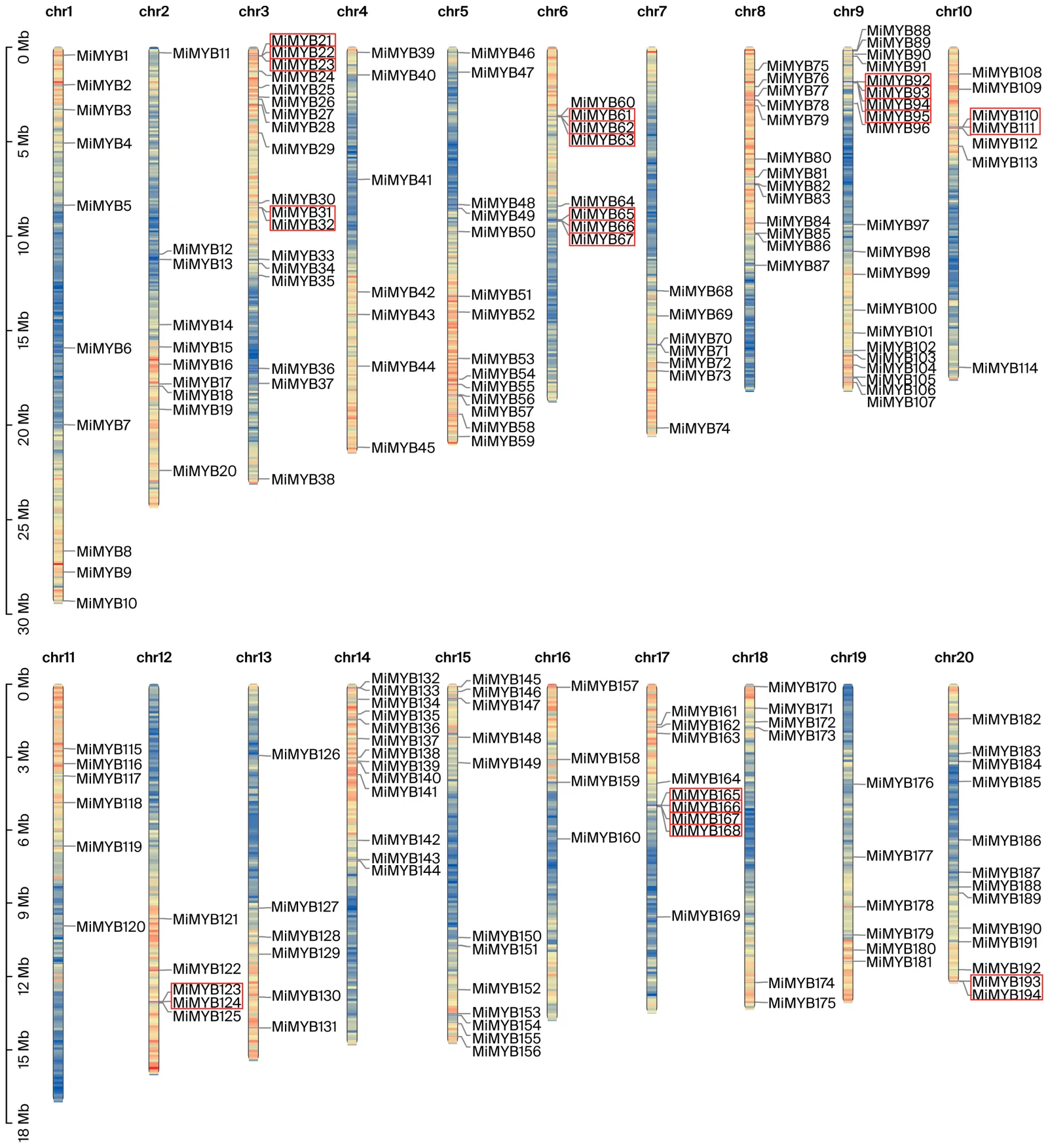
Chromosomal distribution of *MiR2R3-MYB* genes in *M. indica*. The tandem duplicate gene pairs are indicated by the red boxes. Colors indicate the density of chromosome features.

A genome-wide survey revealed that *MiR2R3-MYB* genes were unevenly distributed across the 20 chromosomes (Chr1–Chr20). Chr9 harbored the largest number of *MiR2R3-MYB* genes (20), whereas Chr16 harbored the fewest (4). In total, 16 pairs of tandemly duplicated genes were identified on seven chromosomes: Chr3, Chr6, Chr9, Chr10, Chr12, Chr17, and Chr20. Chr6 contained the largest number of tandemly duplicated gene pairs (four), followed by Chr3, Chr9, and Chr17, each with three pairs. Chr10, Chr12, and Chr20 each contained one pair (Figure 1; Table S3).

### 2.2. Phylogenetic Analysis of MiR2R3-MYB Gene Family

**Figure 2:**
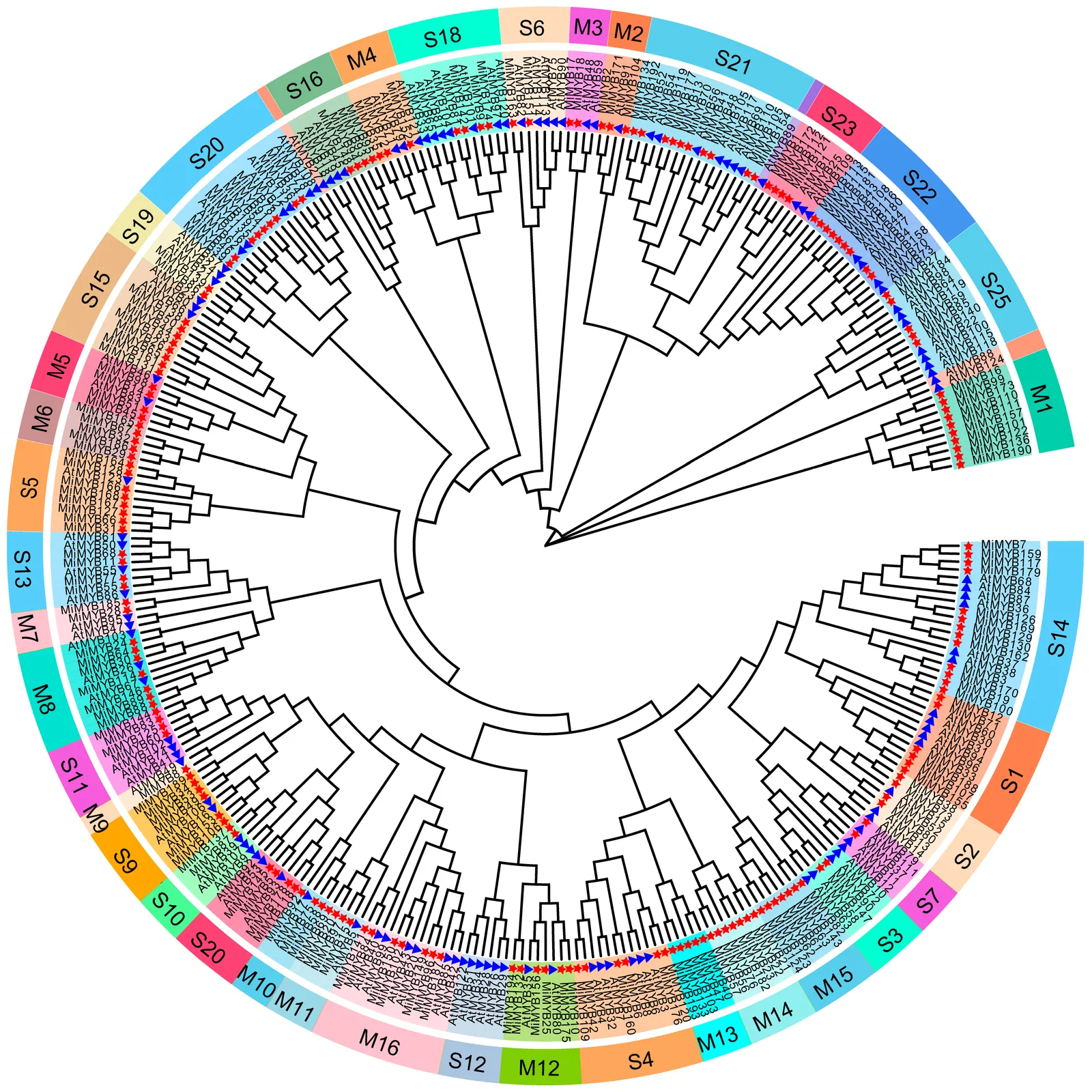
Phylogenetic tree of the *R2R3-MYB* genes from *M. indica* (★) and *A. thaliana* (▲). Colors indicate the corresponding subfamilies.

To investigate the evolutionary relationships within the *R2R3-MYB* gene family, a neighbor-joining phylogenetic tree was constructed in MEGA11 using 194 MiR2R3-MYB protein sequences and 125 R2R3-MYB protein sequences from *A. thaliana* (Table S4). Based on the phylogenetic relationships and bootstrap support values (>50%), the family was classified into 39 subfamilies: S1–S7, S9–S16, S18–S25, and M1–M16 [16]. Subfamily S14 contained the most members (19), whereas subfamily M9 contained only two. No MiR2R3-MYB genes were assigned to subfamily S12, whereas subfamilies M1, M6, M13, M14, and M15 contained only *M. indica* genes (Figure 2). These results suggest that, although the *R2R3-MYB* gene family shares a common evolutionary origin, it has undergone lineage-specific diversification in mango and *A. thaliana*.

### 2.3. Conserved Gene Structure and Motif Analysis of MiR2R3-MYB Genes

**Figure 3:**
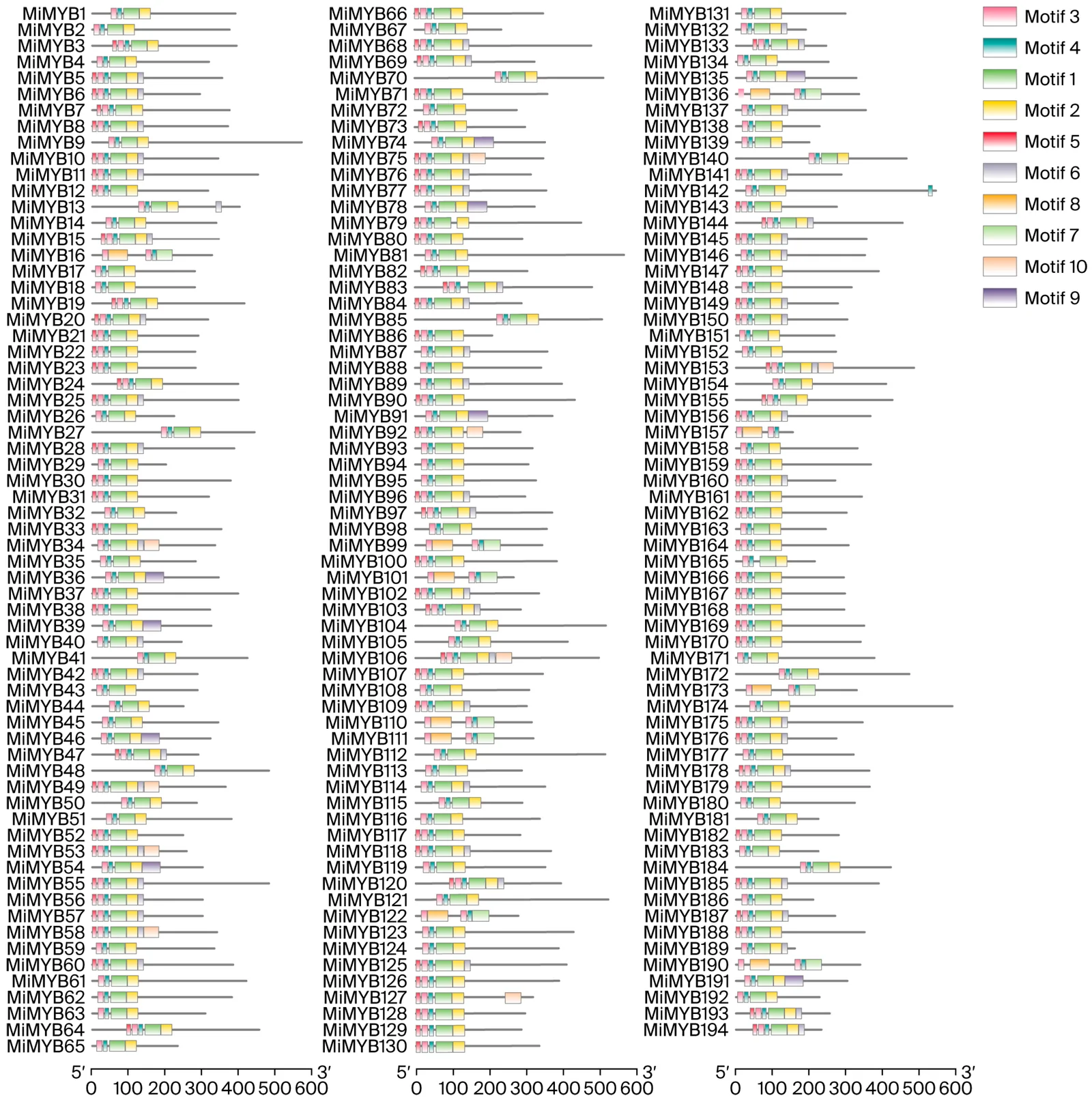
Conserved motif analysis of MiR2R3-MYB proteins. Motifs 1–10 are represented by boxes of different colors, and the scale is shown at the bottom.

The MEME tool was used to identify conserved motifs in MiR2R3-MYB protein sequences. In the analysis, we employed a zero-order background model and the ZOOPS model, with a maximum of 10 motifs and a motif-length range of 6–50 amino acids. All MiR2R3-MYB proteins contained motifs 3 and 4. The most prevalent motif combination comprised motifs 1, 2, 3, and 4 and occurred in 67% of the proteins. The combinations of motifs 5, 3, 4, 1, and 2 and of motifs 5, 3, 4, 1, 2, and 6 occurred in 46% and 41% of the proteins, respectively (Table S5). Motifs 3 and 4 were predominantly located at the N-terminus and contained the conserved tryptophan residues characteristic of the R2 and R3 DNA-binding domains. Motif 7 was specific to subfamily M1, except in MiMYB157. Motif 9 was characteristic of subfamily S20, except in MiMYB36, MiMYB46, and MiMYB39. Motif 10 was restricted to subfamilies S9 and M10, except for MiMYB34 (Figure 3). The conserved and subfamily-specific motif distributions indicate structural diversification among MiR2R3-MYB proteins. Proteins containing unique motifs may therefore have distinct regulatory functions.

**Figure 4:**
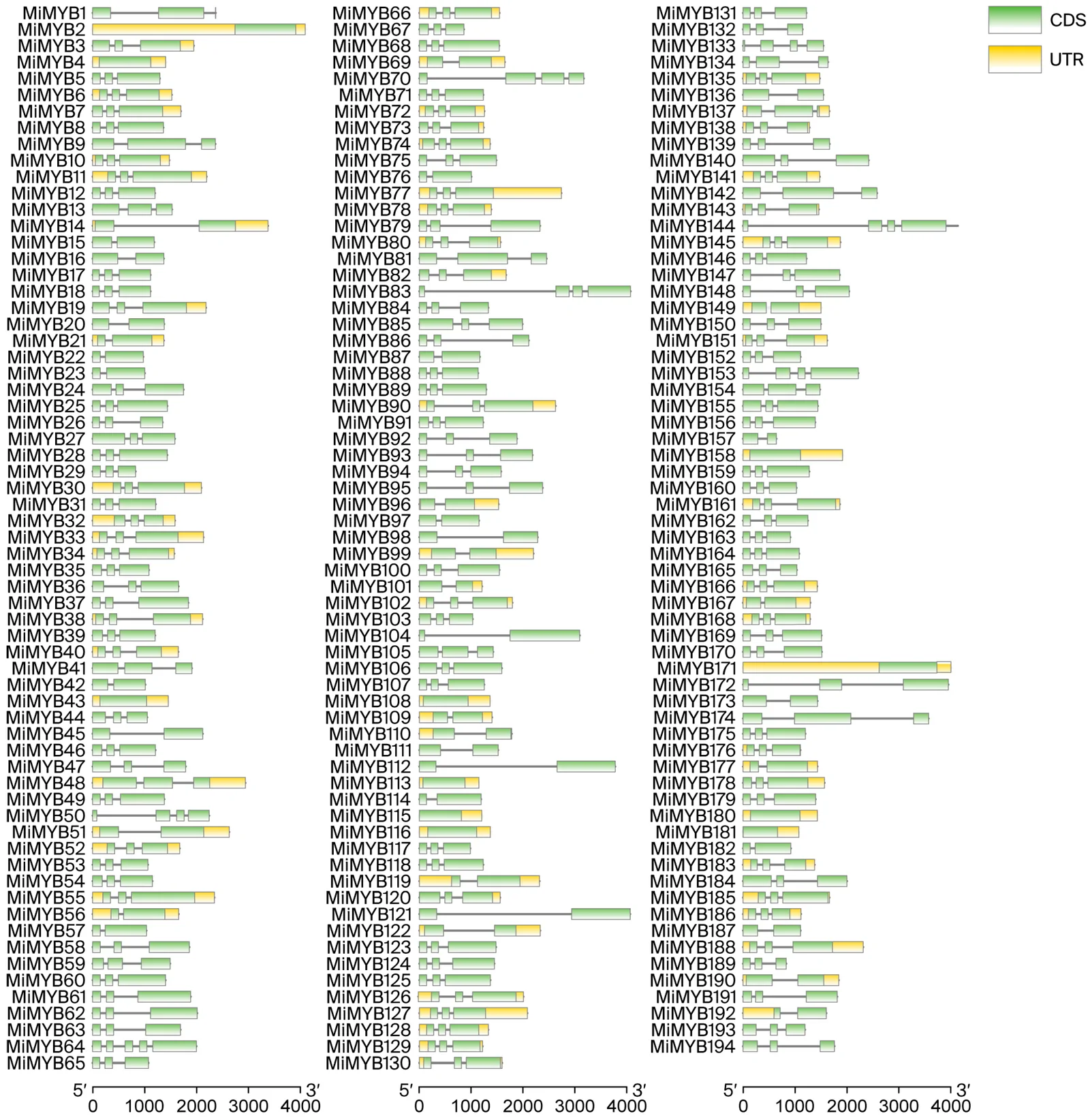
Gene structure of *MiR2R3-MYB* genes. Green boxes represent coding sequence (CDS), yellow boxes represent untranslated region (UTR), and gray lines indicate introns. The scale bar is shown at the bottom.

Gene structure analysis showed that *MiR2R3-MYB* genes contained one to five exons, with *MiMYB64* having the most (five). Of the 194 genes analyzed, 136 (70.1%) contained three exons (Figure 4). Genes within the same subfamily generally had similar exon numbers and lengths, suggesting that gene structure has been conserved within subfamilies during evolution.

**Figure 5:**
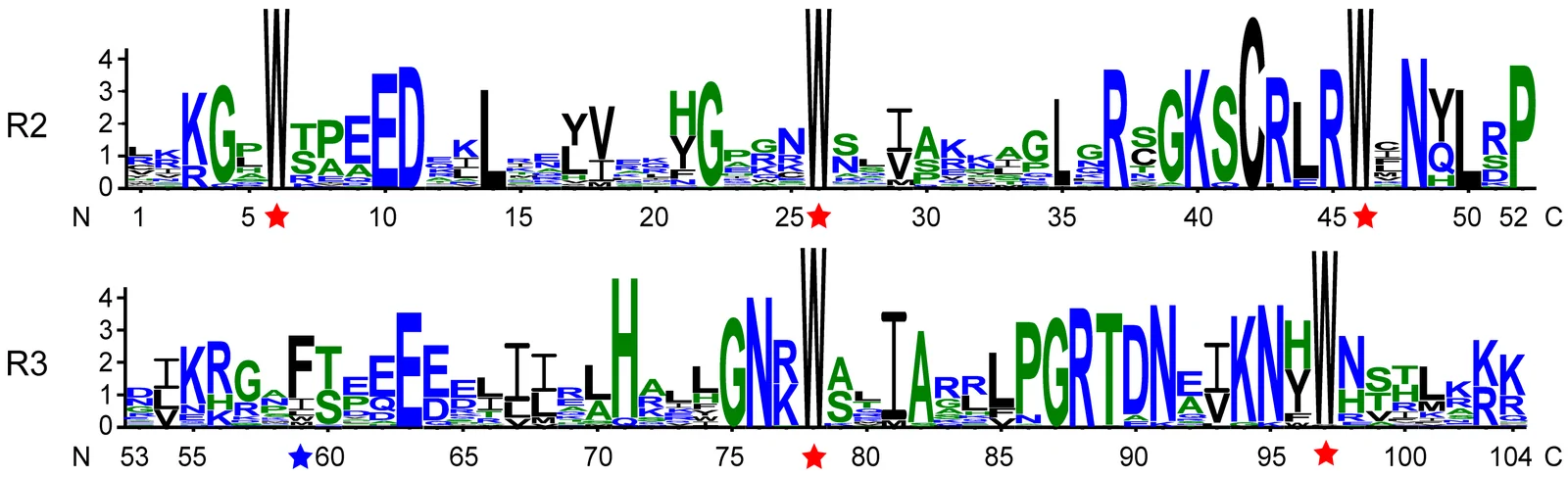
Sequence logos showing the conserved R2 and R3 repeats of the MiR2R3-MYB domain. Red asterisks indicate conserved tryptophan residues (Trp), whereas blue asterisks indicate the amino acid residues that replace tryptophan in the R3 repeat. Different colored letters represent different amino acid properties.

WebLogo analysis was performed to examine conserved residues in MiR2R3-MYB proteins (Figure 5). The conserved tryptophan residues were generally separated by 19 amino acids, a characteristic feature of the R2R3-MYB DNA-binding domain. All three tryptophan residues were conserved in the R2 domain, whereas in the R3 domain, the second and third tryptophans were conserved, while the first was typically replaced by phenylalanine. The R2 domain is responsible for DNA binding, whereas the R3 domain mediates interactions with bHLH cofactors. This conserved pattern supports the potential interaction of MiR2R3-MYB proteins with bHLH transcription factors and their possible involvement in regulating anthocyanin structural genes, suggesting a role in anthocyanin biosynthesis [17].

### 2.4. Analysis of Promoter Cis-Acting Elements in MiR2R3-MYB Genes

PlantCARE analysis of the promoter sequences of *MiR2R3-MYB* genes identified cis-acting elements associated with light response, phytohormone signaling, stress response, and plant growth in 194, 191, 183, and 135 genes, respectively. Light-responsive elements showed the greatest diversity, with 2573 elements. The most abundant were Box 4 (839), G-box (427), and GT1-motif (262), followed by TCT-motif (171) and GATA-motif (117) (Table 1 and Table S6, Figure S1). Stress-responsive elements comprised seven types associated with anaerobic induction, anoxia-specific inducibility, defense, drought inducibility, low-temperature responses, wound responses, and elicitor-mediated activation. The most abundant were ARE (369), MBS (121), and TC-rich repeats (91). Six types of growth-related elements were associated with cell-cycle regulation, palisade mesophyll differentiation, meristem expression, seed-specific regulation, zein metabolism, and circadian control. The predominant elements were the O2-site (95), CAT-box (82), and RY-element (23). Phytohormone-related elements were associated with responses to abscisic acid, auxin, gibberellin, methyl jasmonate, and salicylic acid, as well as endosperm regulation and flavonoid biosynthesis. The most abundant were ABRE (385), CGTCA motif (229), and TGACG motif (225). Variations in cis-element profiles among family members may indicate functional diversification among the *MiR2R3-MYB* genes.

### 2.5. Collinearity, Ka/Ks, and Ortholog Analyses of the MiR2R3-MYB Gene Family

**Figure 6:**
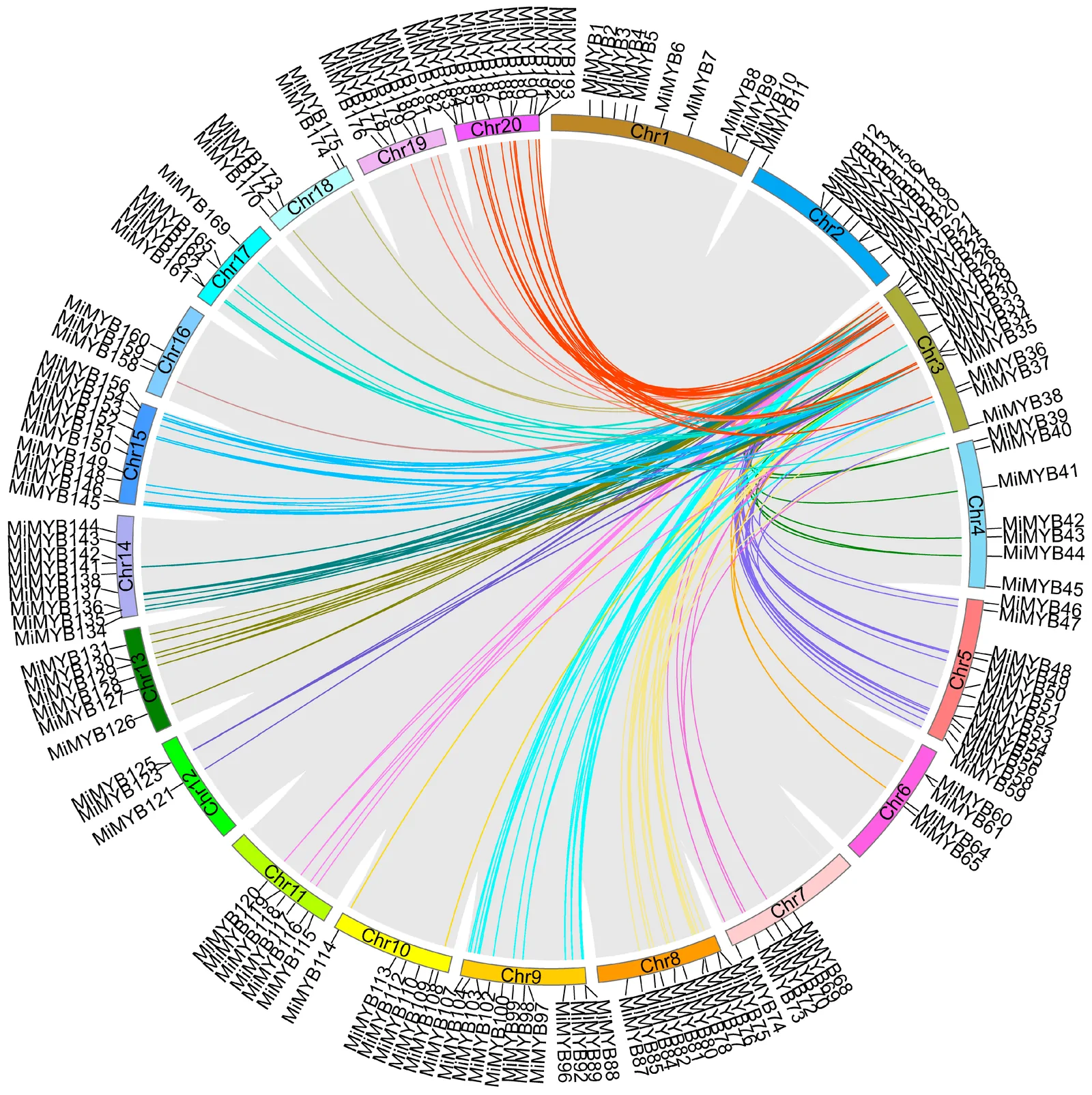
Intragenomic collinearity analysis of the *MiR2R3-MYB* gene family in *M. indica*. Gray lines in the background indicate syntenic blocks across the genome, while colored lines highlight collinear *MiR2R3-MYB* gene pairs located on chromosome 3.

MCScanX was used to identify duplication events within the *MiR2R3-MYB* gene family. In total, 1274 intragenomic collinear gene pairs were identified (Table S7). Chromosome 3 contained the largest number of segmentally duplicated gene pairs, with 248 pairs linking *MiR2R3-MYB* genes on chromosome 3 to those located on other chromosomes. The collinear gene pairs involving chromosome 3 are shown in Figure 6. The *Ka/Ks* ratios of the 248 gene pairs were subsequently calculated, and all values were <0.5 (Table S8), indicating that the duplicated *MiR2R3-MYB* genes were predominantly subject to purifying selection.

**Figure 7:**
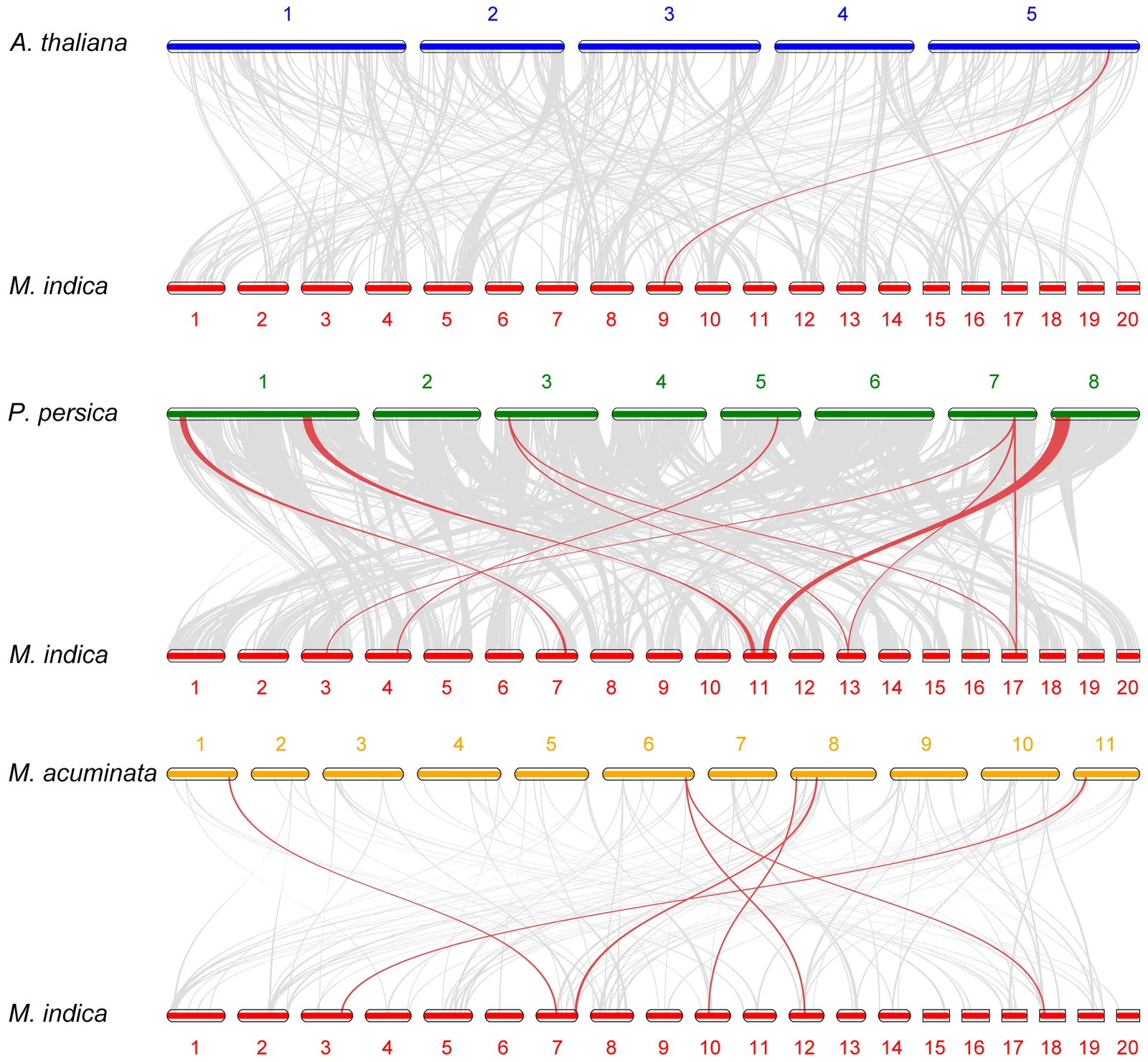
Intergenomic collinearity analysis of *R2R3-MYB* genes between *M. indica* and three representative plant species: *A. thaliana*, *P. persica*, and *M. acuminata*. Red lines highlight collinear blocks containing *R2R3-MYB* gene pairs, whereas gray lines in the background represent syntenic relationships among the other genes in the compared genomes.

To assess the evolutionary relationships of the *MiR2R3-MYB* gene family, intergenomic collinearity analyses were performed between mango and three representative plant species: the eudicots *A. thaliana* and *P. persica*, and the monocot, *M. acuminata*. The *MiR2R3-MYB* genes of *M. indica* shared one, six, and nine syntenic counterparts in *A. thaliana*, *M. acuminata*, and *P. persica*, respectively (Figure 7). Orthologous analysis identified 74 gene pairs between *M. indica* and *P. persica*, 37 pairs between *M. indica* and *M. acuminata,* and 40 pairs between *M. indica* and *A. thaliana* (Table S9). Thus, *M. indica* and *P. persica* exhibited the highest numbers of both syntenic counterparts and orthologous gene pairs among the three comparisons.

### 2.6. Transcriptomic Analysis of MiR2R3-MYB DEGs in Red, Yellow, and Green Mango Peel

**Figure 8:**
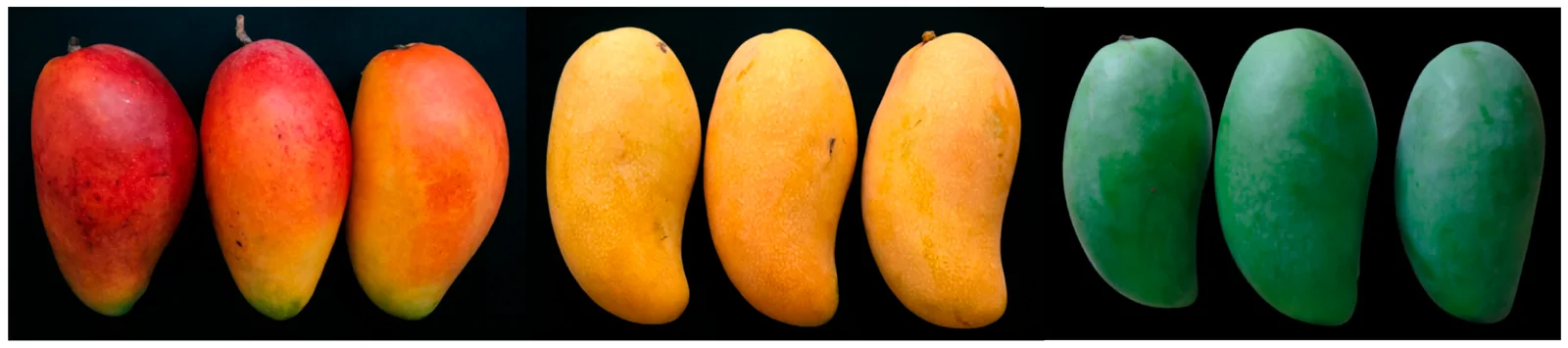
Phenotypic comparison of mango cultivars with different peel colors. From left to right: the red-peel cultivar ‘GF’, the yellow-peel cultivar ‘JH’, and the green-peel cultivar ‘QP’.

**Figure 9:**
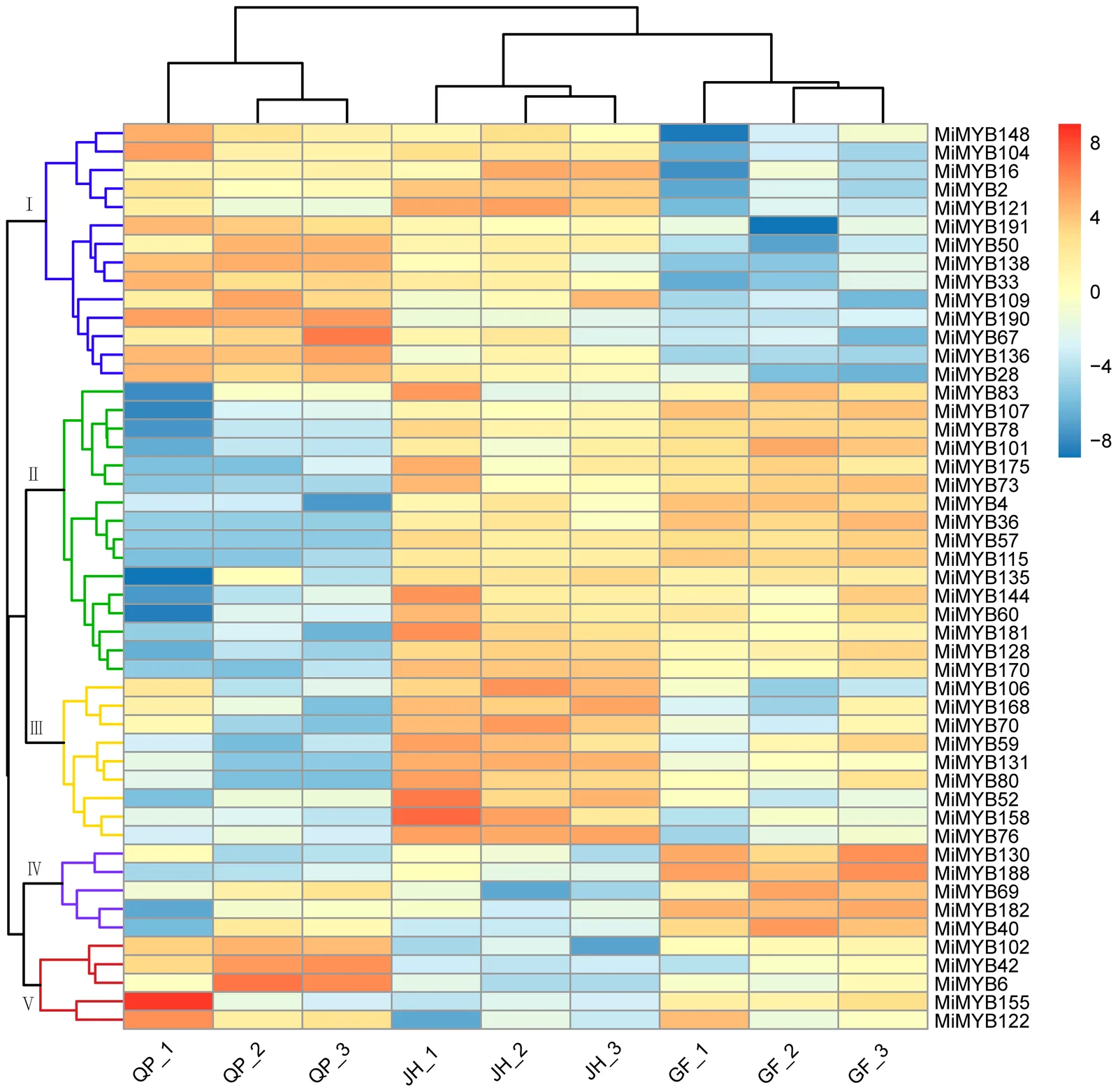
Hierarchical clustering heatmap of 49 *MiR2R3-MYB* DEGs. The heatmap shows log_2_-transformed FPKM values from three biological replicates of the mango cultivars ‘GF’, ‘JH’, and ‘QP’. The color scale ranges from blue (low expression) to red (high expression).

To investigate the molecular basis of peel coloration, we performed transcriptome sequencing on ripe peel samples from three field-grown mango cultivars with distinct peel colors: ‘Guifei’ (red, ‘GF’), ‘Jinhuang’ (yellow, ‘JH’), and ‘Qingpi’ (green, ‘QP’) (Figure 8). Three biological replicates were prepared for each cultivar, generating a total of 69.66 Gb of clean sequencing data from nine samples. Principal component analysis (PCA; Figure S2) showed that the biological replicates clustered together, indicating the high quality and reliability of the sequencing data (Tables S10 and S11). DEGs were identified using the thresholds FDR < 0.05 and |log_2_(fold change)| ≥ 1. Among these DEGs, *MYB*-annotated genes were further screened based on their domain composition to identify *R2R3-MYB* genes. In total, 49 *MiR2R3-MYB* DEGs were identified. Transcript abundance was quantified using FPKM values, and the expression patterns of these genes were visualized in a heatmap (Figure 9; Table S12). Based on their expression profiles, the DEGs were classified into five clusters (I–V). Cluster I DEGs were expressed at higher levels in ‘JH’ and ‘QP’ than in ‘GF’, whereas Cluster II DEGs showed higher expression in ‘JH’ and ‘GF’ than in ‘QP’. Cluster III DEGs were predominantly expressed in ‘JH.’ Cluster IV DEGs showed the highest expression in ‘GF’, whereas Cluster V DEGs were more highly expressed in ’QP’ than in ‘JH’ and ‘GF’. These cultivar-specific expression patterns suggest that *MiR2R3-MYB* DEGs may play distinct roles in mango peel color.

### 2.7. Transcriptomic Analysis of Anthocyanin Structural DEGs and Prediction of MYB-Binding Cis-Elements in Their Promoters

**Figure 10:**
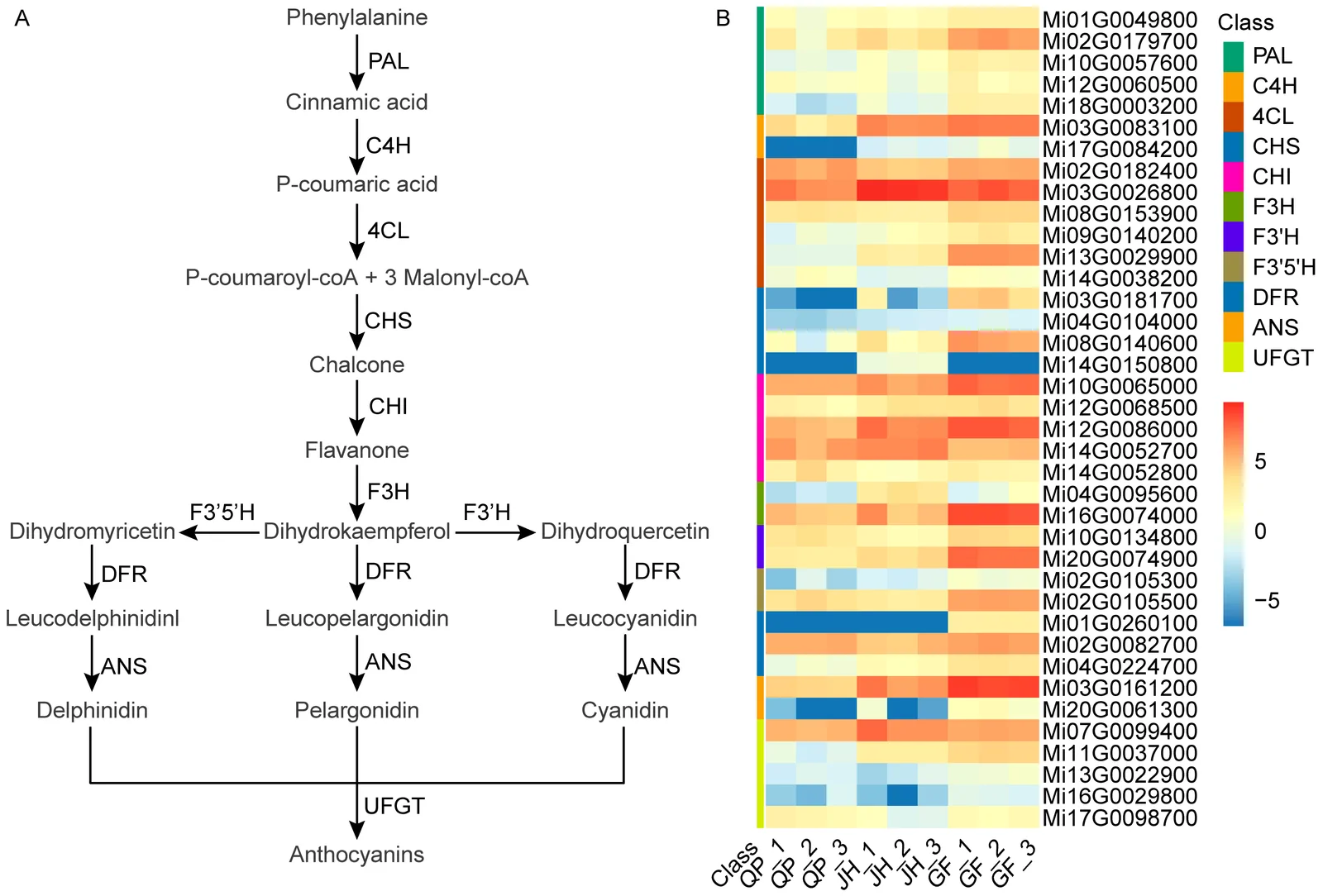
Anthocyanin biosynthetic pathway and hierarchical clustering heatmap of 38 anthocyanin structural DEGs involved in anthocyanin biosynthesis. (**A**) Anthocyanin biosynthetic pathway. (**B**) Hierarchical clustering heatmap of the 38 anthocyanin structural DEGs. The heatmap shows log_2_-transformed FPKM values from three biological replicates of each mango cultivar: ‘GF’, ‘JH’, and ‘QP’.

The expression of anthocyanin structural DEGs was compared among three mango cultivars with different peel colors. Transcriptome analysis of ripe peel samples from red ‘GF’, yellow ‘JH’, and green ‘QP’ identified 38 DEGs encoding structural enzymes in the anthocyanin biosynthesis pathway. Of these, 13 belonged to the upstream phenylpropanoid pathway, comprising five *PAL*, two *C4H*, and six *4CL* genes; 15 belonged to the early flavonoid pathway, comprising four *CHS*, five *CHI*, two *F3H*, two *F3′H*, and two *F3′5′H* genes; and 10 belonged to the late anthocyanin-specific pathway, comprising three *DFR*, two *ANS*, and five *UFGT* genes. Their expression patterns are shown in the heatmap (Figure 10; Table S13). Most of these DEGs were more highly expressed in red ‘GF’ than in green ‘QP’ or yellow ‘JH’ peels. Several genes exhibited similar expression patterns, suggesting their coordinated involvement in anthocyanin biosynthesis. Notably, the late-pathway genes *DFR*, *ANS*, and *UFGT* were more highly expressed in red peels, potentially contributing to cultivar differences in anthocyanin accumulation and peel color.

To investigate the transcriptional regulatory relationships between *MiR2R3-MYB* genes and structural genes in the anthocyanin biosynthetic pathway, we used the JASPAR database to predict MYB-binding sites in the promoter regions of anthocyanin structural DEGs identified in the fruit peels of three mango cultivars with distinct peel colors. In total, 143 putative MYB-binding sites were identified. *PAL* promoters contained the most sites (27), followed by *F3H* (20); *4CL*, *CHS*, and *CHI* (15 each); *F3′5′H* and *UFGT* (13 each); and *ANS* (9). *C4H* and *DFR* promoters contained the fewest sites, with eight each (Table S14).

### 2.8. Analysis of DAMs in Mango Cultivars with Red, Yellow, and Green Peels

**Figure 11:**
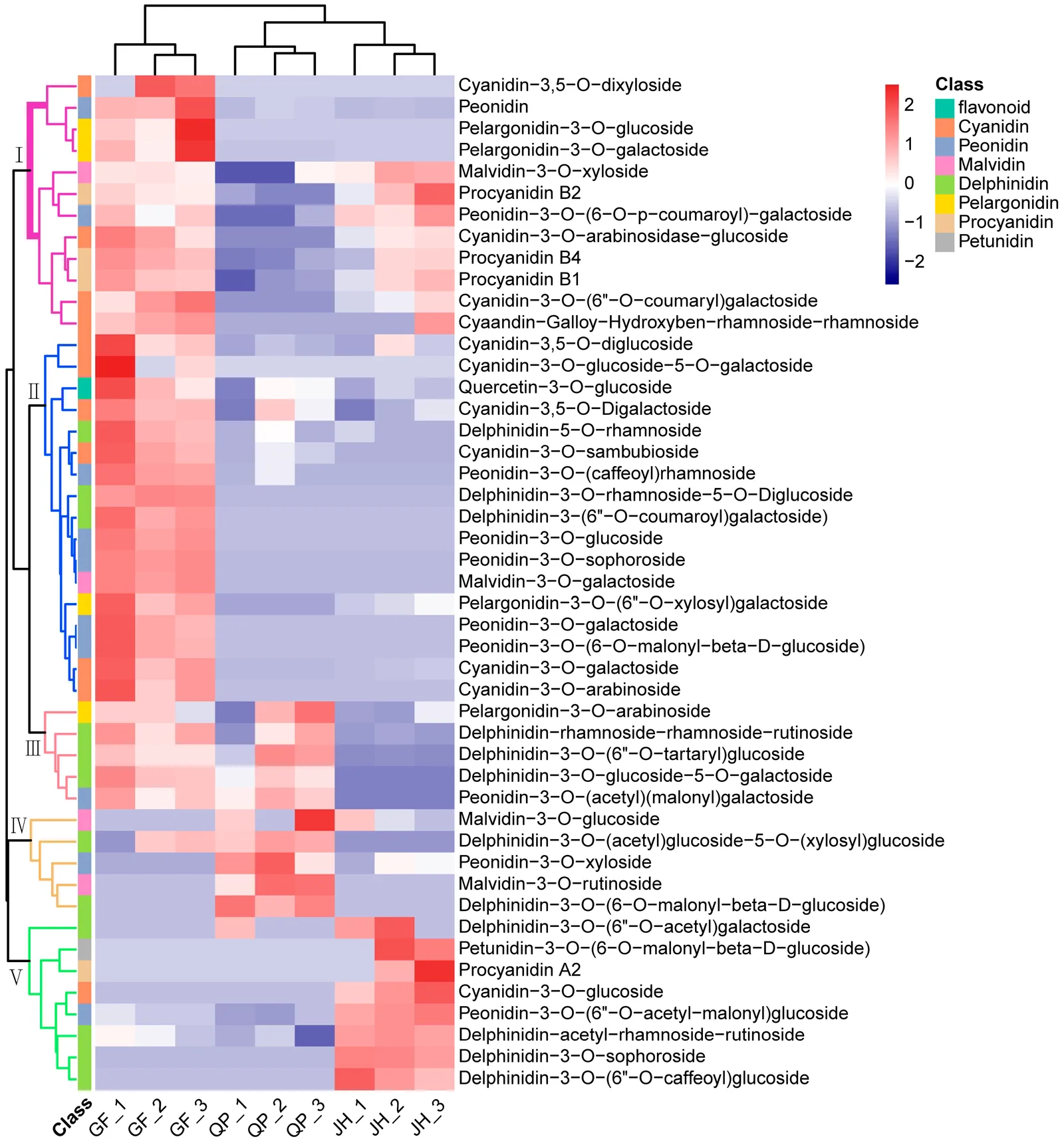
Hierarchical clustering heatmap of DAMs in three mango cultivars (‘GF’, ‘JH’, and ‘QP’), each represented by three biological replicates. Colors indicate Z-score-standardized relative abundance, with red and blue representing high and low abundance, respectively. The color scale ranges from purple (low expression) to red (high expression).

To investigate anthocyanin differences associated with mango peel coloration, targeted anthocyanin profiling was performed on three cultivars with distinct peel colors: red-peel ‘GF’, yellow-peel ‘JH’, and green-peel ‘QP’. Eight metabolite classes were systematically identified: cyanidins, delphinidins, malvidins, pelargonidins, peonidins, petunidins, procyanidins, and other flavonoids (Figure 11). Orthogonal partial least-squares discriminant analysis (OPLS-DA) was used to identify differentially accumulated metabolites (DAMs) among the three cultivars. Using an absolute log_2_ fold change of ≥1, 47 DAMs were identified. Among these, 42 were anthocyanins, accounting for 89.36% of all DAMs. Delphinidins represented the largest proportion of anthocyanins (25.5%), followed by cyanidins (23.4%), peonidins (21.3%), malvidins (8.5%), pelargonidins (8.5%), and petunidins (2.1%). The remaining DAMs comprised proanthocyanidins (8.5%) and other flavonoids (2.1%) (Table S15).

The 47 DAMs were classified into five clusters: Clusters I and II comprised 29 DAMs that accumulated at significantly higher levels in the red-peel cultivar ‘GF’ than in the yellow-peel cultivar ‘JH’ and green-peel cultivar ‘QP’. Notably, peonidin-3-O-glucoside accumulated to a high level in ‘GF’, with an average content of 553.58 μg/g, but was not detected in either ‘JH’ or ‘QP’. The content of pelargonidin-3-O-galactoside in ‘GF’ was 41.84 μg/g, compared with less than 1 μg/g in both ‘QP’ and ‘JH’. In addition, the content of cyanidin-3-O-galactoside in ‘GF’ was 33.60 μg/g, representing 181-fold and 15-fold higher levels than those detected in ‘QP’ and ‘JH’, respectively. DAMs in Cluster III accumulated to higher levels in both ‘GF’ and ‘QP’ than in ‘JH’. In particular, the levels of delphinidin-3-O-(6″-O-tartaryl) glucoside in ‘GF’ and ‘QP’ were 9.81-fold and 10.91-fold higher, respectively, than that in ‘JH’. DAMs in Clusters IV and V generally accumulated at low levels and exhibited only minor differences among the three cultivars. Overall, these results demonstrate that anthocyanin accumulation patterns vary substantially among mango cultivars with distinct peel-color phenotypes.

### 2.9. Correlation Analysis and Network Construction Between MiR2R3-MYB DEGs and Anthocyanin DAMs

Pearson correlation analysis was performed across all samples to evaluate the relationships between the *MiR2R3-MYB* DEGs and anthocyanin DAMs. Pearson correlation coefficients were calculated using the cor function in R, and correlations were considered significant at |r| > 0.8 and *p*-value < 0.05. Among the 49 *MiR2R3-MYB* DEGs identified by transcriptomic analysis, 45 were significantly correlated with 36 anthocyanin DAMs. Specifically, 40 *MiR2R3-MYB* DEGs were positively correlated with 29 anthocyanin DAMs, whereas 35 *MiR2R3-MYB* DEGs were negatively correlated with 31 anthocyanin DAMs. Notably, 30 *MiR2R3-MYB* DEGs and 24 anthocyanin DAMs were shared between the positive- and negative-correlation groups, indicating that a single gene can be positively correlated with some anthocyanin DAMs but negatively correlated with others (Table S16; Figure S3).

**Figure 12:**
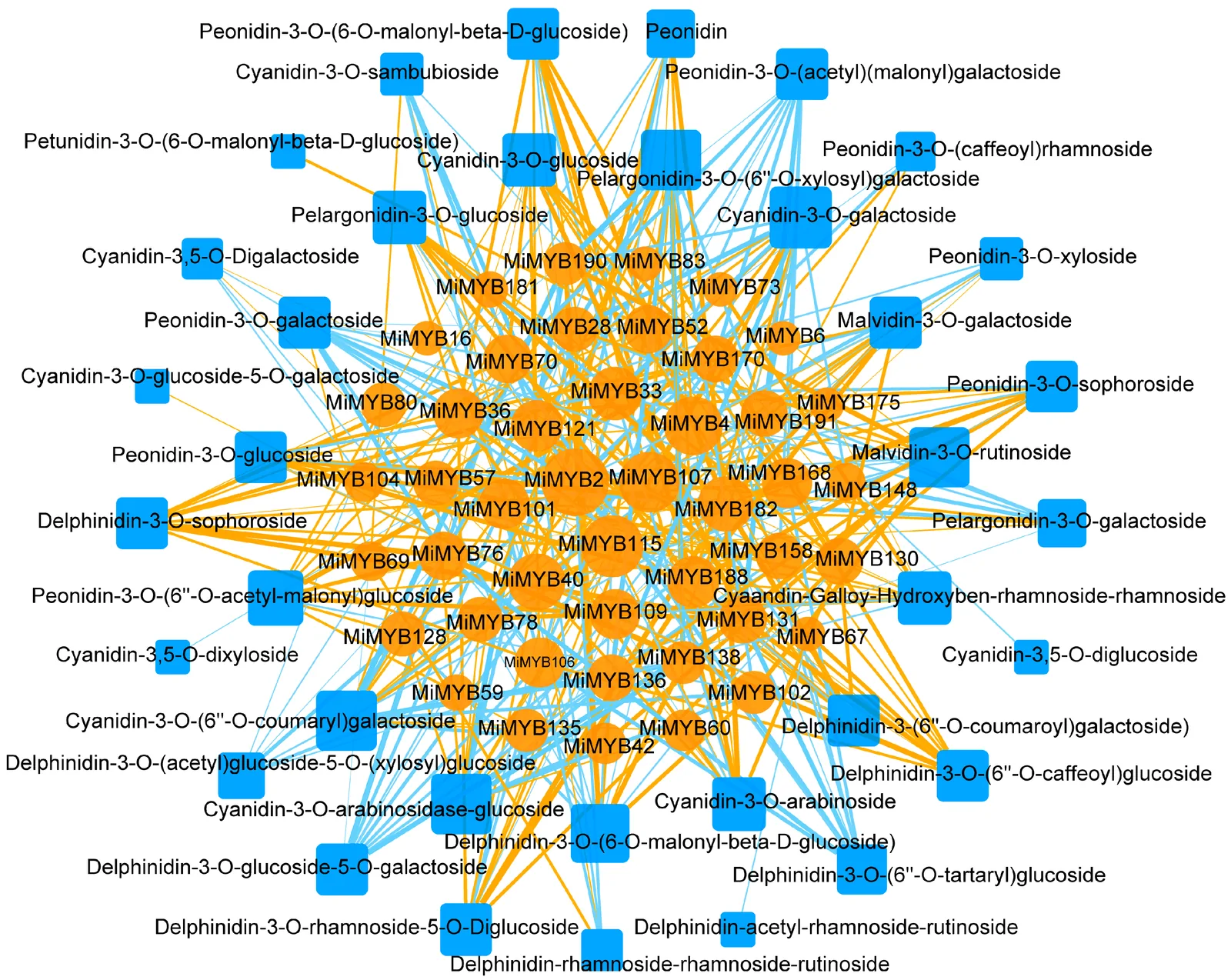
Correlation network between 45 *MiR2R3-MYB* DEGs and 36 anthocyanin DAMs. Orange circles represent genes, and blue squares represent metabolites. Node size is proportional to the number of connections: larger circles indicate genes associated with more anthocyanins, while larger squares indicate anthocyanins associated with more genes. Edge width is proportional to the absolute correlation coefficient, with thicker edges indicating stronger correlations.

To visualize the correlations between *MiR2R3-MYB* DEGs and anthocyanin DAMs, a correlation network was also constructed based on Pearson correlation coefficients. The network comprised 81 nodes (45 gene nodes and 36 anthocyanin nodes) connected by 345 edges. Orange circles represent genes and blue squares represent anthocyanins. Node size is proportional to the number of connections: larger circles indicate genes correlated with more anthocyanins, whereas larger squares indicate anthocyanins correlated with more genes. Edge thickness reflects the absolute strength of the correlation, with thicker edges indicating stronger absolute correlations. Notably, *MiMYB2*, *MiMYB115*, and *MiMYB107* were among the most highly connected genes in this network. *MiMYB2* showed the highest connectivity, with 18 negative and two positive correlations. *MiMYB115* and *MiMYB107* showed the next highest connectivity: *MiMYB115* was correlated with 17 anthocyanins (14 positive and three negative correlations), whereas *MiMYB107* was correlated with 16 anthocyanins (15 positive and one negative correlation). Among all *MiR2R3-MYB* genes, *MiMYB107* was positively correlated with the largest number of anthocyanins (Figure 12; Table S16).

### 2.10. RT-qPCR Validation of Eight Selected Candidate MiR2R3-MYB DEGs

**Figure 13:**
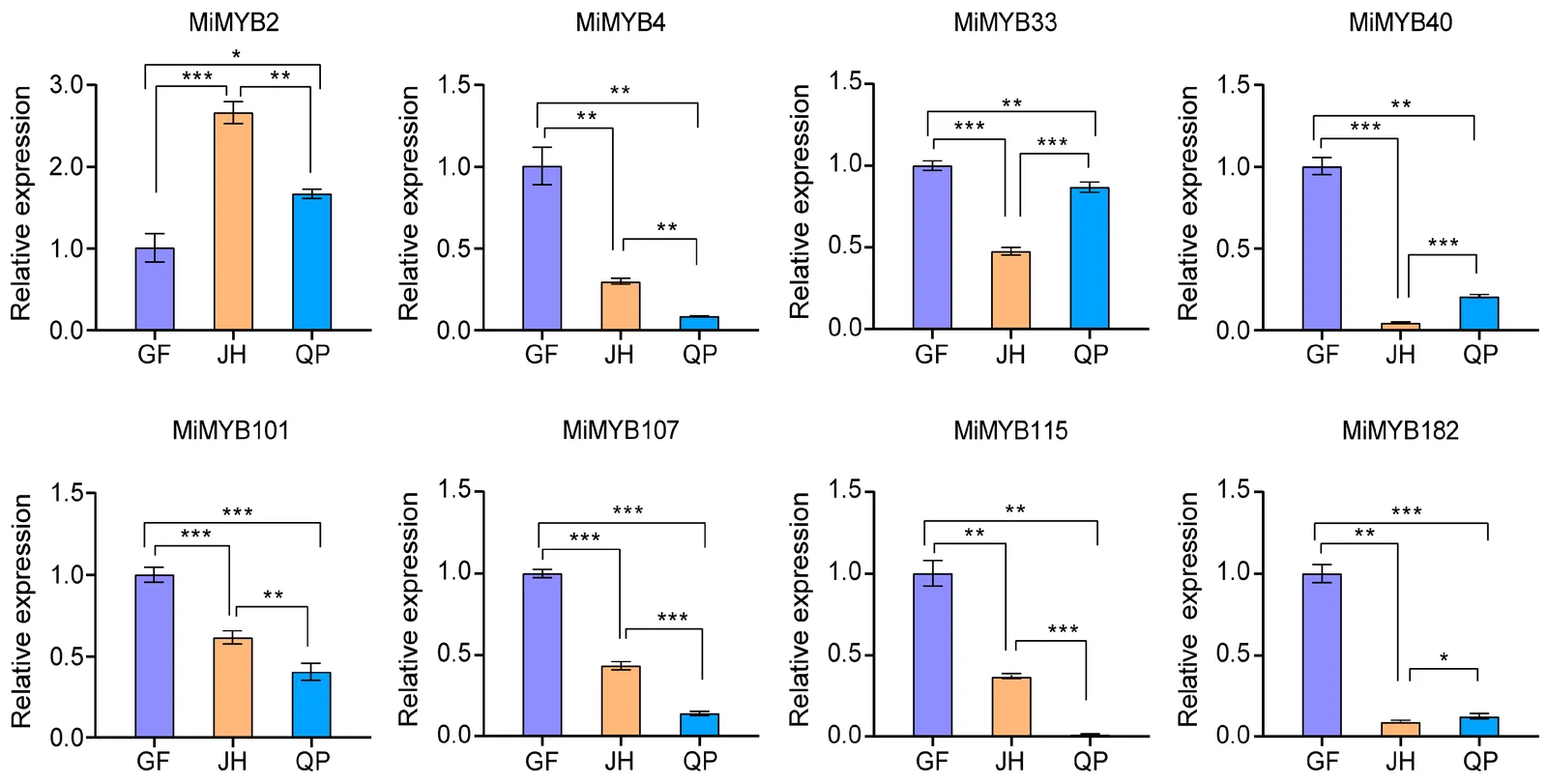
RT–qPCR validation of the relative expression levels of eight *MiR2R3-MYB* candidate genes. Bar charts show the mean relative expression levels of these genes in the peels of red ‘GF’, yellow ‘JH’, and green ‘QP’ mango cultivars. Error bars represent the standard deviation (SD) of three biological replicates. Asterisks indicate significant differences based on Student’s *t*-test. (* *p* < 0.05, ** *p* < 0.01, and *** *p* < 0.001).

To validate the expression patterns of central genes in the network, eight *MiR2R3-MYB* DEGs were selected for RT–qPCR analysis based on their numerous significant correlations with anthocyanin metabolites. Gene-specific primers were designed based on the coding sequences of the selected genes (Table S17). Three biological replicates were analyzed for each cultivar. The reliability of the RT–qPCR results was supported by RNA quality assessment (Figure S4), typical single-peak melting curves for all tested genes (Figure S5). *MiMYB2* showed the highest expression in the peel of the yellow cultivar ‘JH’, with significantly higher expression than in the red cultivar ‘GF’ and the green cultivar ‘QP’. In contrast, *MiMYB4*, *MiMYB33*, *MiMYB40*, *MiMYB101*, *MiMYB107*, *MiMYB115*, and *MiMYB182* showed the highest expression in ‘GF’, with significantly higher expression than in ‘JH’ and ‘QP’ (Figure 13). The RT–qPCR results showed expression trends consistent with those obtained from the transcriptomic analysis for all eight candidate *MiR2R3-MYB* DEGs, although differences in the magnitude of expression changes were observed.

### 2.11. Subcellular Localization of theMiMYB107-eGFP Fusion Protein

**Figure 14:**
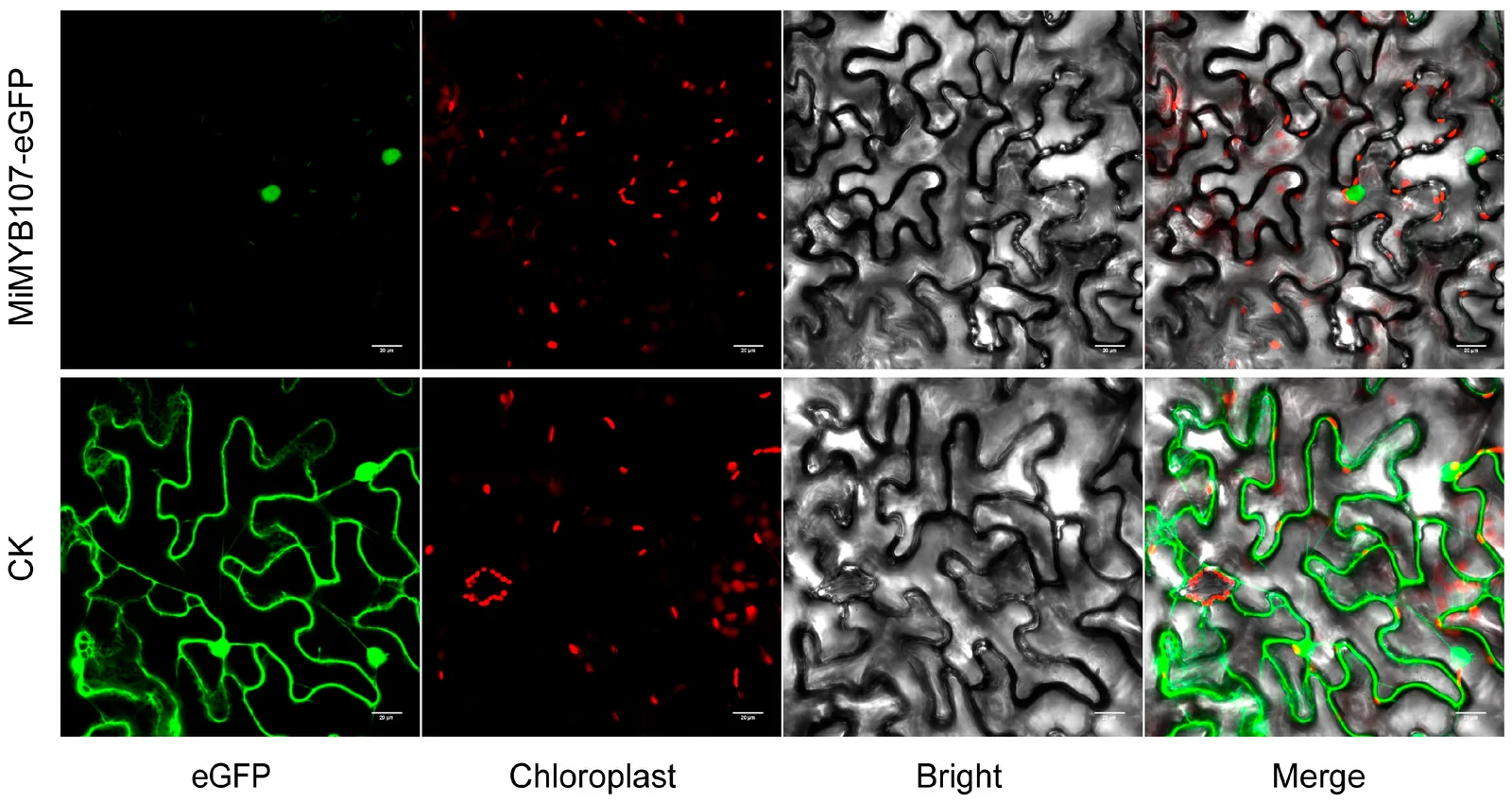
Subcellular localization of the MiMYB107-eGFP fusion protein in epidermal cells of *N. benthamiana* leaves. Free eGFP was used as a control and imaged under identical confocal settings. GFP, green fluorescent protein; Chloroplast, chloroplast autofluorescence; Bright, bright field; Merge, merge field. Scale bars = 20 μm.

Subcellular localization analysis predicted that all MiR2R3-MYB proteins were localized to the nucleus. Because *MiMYB107* showed the greatest number of positive correlations with anthocyanin metabolites, it was selected for experimental validation of its subcellular localization. The MiMYB107-eGFP fusion construct was transiently expressed in *Nicotiana benthamiana* leaves, and the subcellular localization of the fusion protein was examined by laser-scanning confocal microscopy. Three independent biological replicates were performed, and representative images are shown (Figure 14 and Figure S6). The MiMYB107-eGFP fluorescence signal was predominantly localized to the nucleus, consistent with the predicted localization.

### 2.12. Western Blot Analysis of the MiMYB107-eGFP Fusion Protein

**Figure 15:**
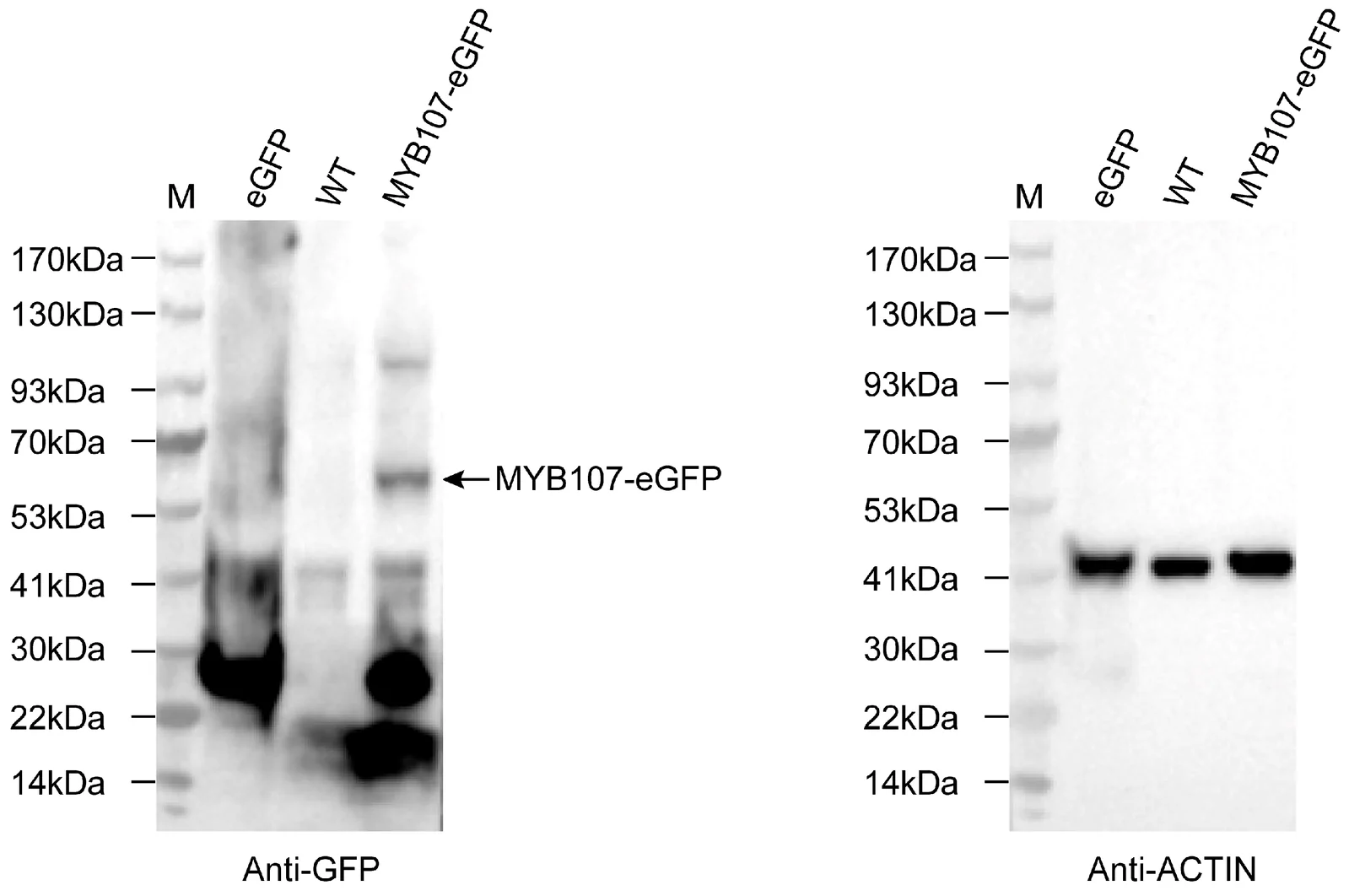
Qualitative Western blot analysis of the MiMYB107-eGFP fusion protein expressed in *N. benthamiana* leaves. Total proteins were extracted and immunoblotted with an anti-GFP antibody. The target fusion protein is indicated at approximately 61.6 kDa. Lanes contain the protein marker (M), free eGFP control, wild-type (WT) control, and MiMYB107-eGFP sample, respectively. ACTIN was detected as a loading control. This blot confirms the specific expression of the MiMYB107-eGFP fusion protein.

To confirm the expression of MiMYB107, the MiMYB107-eGFP fusion protein was transiently expressed in *N. benthamiana* leaves, and protein accumulation was assessed by Western blotting. Chemiluminescence signals were captured using an automated imaging system. The predicted molecular weight of MiMYB107 was 34.6 kDa as calculated using the ExPASy ProtParam tool, while eGFP had a molecular weight of approximately 27 kDa. Thus, the expected molecular weight of the MiMYB107-eGFP fusion protein was approximately 61.6 kDa. Western blotting detected a specific band at approximately 61.6 kDa (Figure 15), consistent with the predicted molecular weight and providing qualitative confirmation of MiMYB107-eGFP fusion-protein expression in *N. benthamiana* leaves.

## 3. Discussion

Peel color strongly affects mango market value and consumer acceptance, with red coloration mainly resulting from anthocyanin accumulation. R2R3-MYB TFs are key regulators of anthocyanin biosynthesis and fruit coloration. Genome-wide analyses have identified 126, 118, 285, and 146 *MYB* genes in *A. thaliana* [16], *P. persica* [18], *M. Acuminata* [19], and *Pyrus ussuriensis* [20], respectively. In mango, only 54 MYB genes had previously been identified [21]. Using a high-quality mango genome assembly, we identified 370 MYB genes, including 171 1R-MYBs, 194 R2R3-MYBs, four 3R-MYBs, and one 4R-MYB. This difference likely reflects improvements in genome annotation, assembly quality, and gene-identification criteria. Previous studies were largely based on earlier genome assemblies, whereas in the present study, we performed a genome-wide search using an updated, high-quality mango genome assembly and current database resources. Given the essential roles of R2R3-MYB TFs in plant development and secondary metabolism, this study focused specifically on the 194 *MiR2R3-MYB* genes identified in mango.

The predicted physicochemical properties of the MiR2R3-MYB proteins varied considerably, with substantial differences in protein length, molecular weight, theoretical isoelectric point, and aliphatic index, indicating marked variation in protein charge and thermal stability. Most MiR2R3-MYB proteins were predicted to be unstable and hydrophilic. This is consistent with the structural characteristics commonly observed in transcription factors, which often function through flexible protein–protein and protein–DNA interactions. These physicochemical differences may influence protein stability, subcellular behavior, and regulatory activity, thereby contributing to the functional diversification of the *MiR2R3-MYB* family in mango. Nevertheless, these predicted properties require experimental validation.

Phylogenetic analysis grouped 194 *MiR2R3-MYB* genes and 125 *A. thaliana R2R3-MYB* genes into 39 subfamilies. *MiR2R3-MYB* genes were distributed among 24 subfamilies previously defined in *A. thaliana*, suggesting that genes within the same clade may share related functions. However, *MiR2R3-MYB* genes were detected only in clades M1-M16, which may indicate lineage-specific diversification and functional divergence in mango. According to previous studies in *Fragaria × ananassa* [22], *Theobroma cacao* [23], and *Medicago truncatula* [24], subfamilies S1, S4, S15, S20, and S22 may be involved in stress responses, phenylpropanoid metabolism and anthocyanin biosynthesis, and cell differentiation, respectively [17]. The mango-specific subfamily M may represent a lineage-specific group, although its function remains unclear. Conserved motif composition and exon–intron organization within subfamilies further supported the phylogenetic classification. All MiR2R3-MYB proteins contained motifs 3 and 4, which were mainly located in the N-terminal R2 DNA-binding domain and may contribute to DNA recognition and interactions with bHLH cofactors. In the R3 domain, the first tryptophan residue was frequently replaced by phenylalanine, a conserved feature that may affect protein–DNA interactions and transcriptional regulation [17,25,26]. Most *MiR2R3-MYB* genes contained three exons and two introns, consistent with previous observations in plant *R2R3-MYB* families [27]. Cis-acting elements in gene promoters are important for transcriptional regulation and can provide clues about gene function. In this study, the promoters of *MiR2R3-MYB* genes contained diverse elements associated with light, stress, growth, and phytohormone responses. Their composition suggests that *MiR2R3-MYB* genes may be regulated by the combined effects of environmental stimuli, hormonal signals, and developmental cues, supporting the potential functional diversification of this gene family.

The *MiR2R3-MYB* genes were unevenly distributed across the chromosomes, and both segmental and tandem duplications were detected. Chromosome 3 contained the largest number of segmentally duplicated gene pairs (248). The *Ka/Ks* ratios of the syntenic gene pairs on chromosome 3 were all below 0.5, indicating that these duplicated genes have mainly evolved under purifying selection and are subject to strong functional constraints. Comparative synteny analysis identified more orthologous and collinear *R2R3-MYB* gene pairs between *M. indica* and *P. persica* than between *M. indica* and either *A. thaliana* or *M. acuminata*. This result indicates a higher degree of syntenic conservation within the *R2R3-MYB* gene family between *M. indica* and *P. persica*. The functional conservation of these syntenic gene pairs remains to be investigated.

Transcriptomic analysis of the mango cultivars ‘GF’, ‘JH’, and ‘QP’ identified 49 *MiR2R3-MYB* DEGs and 38 anthocyanin structural DEGs, suggesting their potential involvement in mango peel-color development. The promoters of these structural genes contained 143 putative MYB-binding sites, providing candidate targets for MYB-mediated transcriptional regulation. Previous studies have shown that MYB proteins can either activate or repress anthocyanin biosynthesis. For example, *SoMYB44* promotes lilac coloration by binding to the promoter of *SoUFGT1* and activating its expression [28], whereas *CmMYB4* represses anthocyanin accumulation by suppressing *CmDFR* and *CmUFGT* [29]. Therefore, differentially expressed *MiR2R3-MYB* genes may regulate mango peel coloration by modulating anthocyanin structural genes, although this hypothesis requires validation through expression correlation, promoter-binding, and transactivation assays.

Metabolomic analysis identified 47 DAMs in mango cultivars, including 42 anthocyanins that accounted for 89.36% of the total. The red-peel cultivar ‘GF’ accumulated significantly higher levels of peonidin-3-O-glucoside, pelargonidin-3-O-galactoside and cyanidin-3-O-galactoside than ‘QP’ and ‘JH’, suggesting that these pigments contribute to red peel coloration. This finding is consistent with reports that peonidin and cyanidin are major pigments in red mango [1], red hawthorn [30], *Rosa rugosa* [31], *Paeonia* [32], and Chinese cherry [33]. In *C. pinnatifida* ‘Ruanzi’, cyanidin-3-O-galactoside accounts for more than 95% of the total peel anthocyanin content [30]. Similarly, red mango cultivars contain higher levels of cyanidin-3-O-galactoside and cyanidin-3-O-glucoside than the green cultivars, further supporting the role of cyanidin derivatives in mango peel coloration.

Correlation analysis identified 45 *MiR2R3-MYB* DEGs significantly associated with 36 anthocyanin DAMs. Individual anthocyanins were correlated positively or negatively with different *MiR2R3-MYB* genes, while a single gene could show contrasting correlations with different anthocyanins. For example, *MiMYB107* was positively correlated with 15 anthocyanins and negatively correlated with one, whereas *MiMYB2* showed positive correlations with two and negative correlations with 18 anthocyanins. Similar associations between *MYB* expression and anthocyanin accumulation have been reported in apple and *Passiflora edulis* [34,35]. These results suggest that *MiR2R3-MYB* genes may participate in the spatiotemporal regulation of mango peel coloration, with positively and negatively correlated genes representing candidate activators and repressors, respectively.

The correlation network comprised 45 *MiR2R3-MYB* DEGs and 36 anthocyanin DAMs, with *MiMYB2*, *MiMYB4*, *MiMYB33*, *MiMYB40*, *MiMYB101*, *MiMYB107*, *MiMYB115*, and *MiMYB182* occupying central positions and showed high connectivity. RT-qPCR validation confirmed expression trends consistent with the transcriptome data, although differences in expression abundance were observed between the two methods. These genes were therefore selected as candidates for subsequent functional analyses. Among them, *MiMYB107* showed positive correlations with 15 anthocyanins, the highest number observed, and localized to the nucleus, supporting its potential role as a transcription factor. Western blotting detected the MiMYB107-eGFP fusion protein at approximately 53–70 kDa, close to its predicted molecular weight of 61.6 kDa. Together, these results identify *MiMYB107* as a promising candidate gene of anthocyanin accumulation in mango, although its function requires further validation.

In this study, we examined only three mango cultivars, and further validation across a wider range of cultivars representing diverse genetic backgrounds is necessary to determine whether our findings are generally applicable to mango germplasm. Moreover, the correlations between *MiR2R3-MYB* expression and anthocyanin accumulation indicate potential regulatory relationships but do not establish direct causation. Future studies should therefore expand the analysis to a larger panel of cultivars with diverse genetic backgrounds, peel colors, and anthocyanin profiles, while also including multiple developmental stages and environmental conditions. In addition, candidate genes such as *MiMYB107* should be functionally validated through in vitro DNA-binding and transactivation assays, as well as transient or stable overexpression and silencing experiments. Integrating transcriptomic, metabolomic, and biochemical evidence will help clarify the regulatory mechanisms by which MiR2R3-MYB transcription factors control anthocyanin biosynthesis and contribute to mango peel coloration.

## 4. Materials and Methods

### 4.1. Genome-Wide Identification, Physicochemical Properties, and Chromosomal Distribution of R2R3-MYB Genes in M. indica

The genome assembly, gene annotation, and protein sequences of the ‘Alphonso’ mango were obtained from NCBI [36], and the HMM profile for the MYB domain (PF00249) was obtained from InterPro 105.0 (https://www.ebi.ac.uk/interpro/) (accessed on 10 December 2025) [37]. To identify members of the *R2R3-MYB* gene family, HMMER3.4 [38] searches against the *M. indica* genome were performed using an E-value ≤ 1 × 10^−5^. Redundant protein sequences were removed manually. The identified *R2R3-MYB* genes were sequentially named based on their chromosomal positions, using the prefix ‘Mi’ (derived from *M. indica*), followed by ‘MYB’ (e.g., MiMYB). The physicochemical properties of the identified MiR2R3-MYB proteins were predicted using ExPASy ProtParam software (http://Web.ExPASy.Org/protparam/) (accessed on 7 February, 2026) [39]. The genomic coordinates of the *MiR2R3-MYB* genes and the lengths of the individual chromosomes were retrieved from the genome annotation files using SAMtools [40]. The chromosomal distribution of *MiR2R3-MYB* genes was visualized using TBtools (v2.468) software [41].

### 4.2. Phylogenetic Analysis of MiR2R3-MYB Genes

The protein sequences encoded by *R2R3-MYB* genes in *A. thaliana* (AtMYB1–AtMYB125) were retrieved from the Phytozome v13 database (https://phytozome-next.jgi.doe.gov/; accessed on 6 March 2025) [42]. The MiR2R3-MYB and *A. thaliana* R2R3-MYB protein sequences were aligned using the ClustalW algorithm implemented in MEGA 11 with default parameters. A neighbor-joining phylogenetic tree was constructed in MEGA 11 using the Poisson correction model with pairwise deletion of gaps, and branch support was assessed with 1000 bootstrap replicates [43]. Based on tree topology, MiR2R3-MYB proteins were assigned to distinct subfamilies according to the previously reported classification of the *A. thaliana R2R3-MYB* gene family [16]. The resulting phylogenetic tree was visualized and annotated using the web-based EvolView platform (https://www.evolgenius.info/evolview/; accessed on 6 March 2026) [44].

### 4.3. Gene Structure and Conserved Motif Analysis of MiR2R3-MYB Genes

The GFF-formatted gene annotation file of *M. indica* was obtained from the NCBI database (https://www.ncbi.nlm.nih.gov/datasets/genome/GCF_011075055.1/; accessed on 14 March 2025) [36], and the R2R3-MYB protein sequences were aligned using multiple alignments using a fast Fourier transform [45]. Conserved motif analysis was conducted using the MEME Suite by specifying a motif width range of 6–50 amino acids and limiting the search to a maximum of 10 motifs (https://meme-suite.org/meme/tools/meme; accessed on 17 January 2026) [46]. The sequence logo of the R2R3 domain was generated by aligning the protein sequences of 194 R2R3-MYB members and visualizing the alignment using WebLogo [47]. The 2000 bp regions upstream of the transcription start sites of the *MiR2R3-MYB* genes were considered putative promoter regions and analyzed for cis-regulatory elements using the PlantCARE database (https://bioinformatics.psb.ugent.be/webtools/plantcare/html/; accessed on 23 November 2025) [48].

### 4.4. Ka/Ks Ratio, Collinearity, and Ortholog Analysis of MiR2R3-MYB Genes

The nonsynonymous substitution rate (*Ka*), synonymous substitution rate (*Ks*), and their ratio (*Ka/Ks*) were calculated using KaKs_Calculator 2.0 [49]. Intra- and intergenomic collinear blocks were identified using MCScanX (v1.0.0) [50] with default parameters, and orthologous relationships were inferred using OrthoFinder (v3.1.5) [51]. These analyses were performed to investigate the evolutionary relationships of the *R2R3-MYB* gene family among *M. indica*, *A. thaliana*, *M. acuminata*, and *P. persica*. Genomic data for *A. thaliana*, *M. acuminata*, and *P. persica* were obtained from the Phytozome v13 database (https://phytozome-next.jgi.doe.gov/; accessed on 6 March 2025) [42].

### 4.5. Plant Materials and Treatments

As an important indicator of the external quality of fruit, peel color largely determines its commercial value [52]. Three mango cultivars with distinct peel colors were selected for this study: ‘GF’, ‘JH’, and ‘QP’. These cultivars were obtained from the germplasm resource collection and grown at the Hainan Provincial Germplasm Resources Garden in Danzhou, Hainan, China. Fruits were harvested at the half-ripe stage (slightly soft) in June 2023 [53]. For each cultivar, fruits of uniform size and without visible mechanical damage or disease symptoms were selected. Three biological replicates were prepared for each cultivar, with each replicate derived from an independent plant. Three fruits were collected from each plant, and their peels were separated and pooled as one biological replicate. The peel samples were immediately frozen in liquid nitrogen and stored at −80 °C until anthocyanin content determination, transcriptomic analysis, and RT–qPCR analysis. To preserve the independence of the biological replicates, each replicate was processed separately throughout tissue collection, RNA extraction, metabolite extraction, library preparation, and sequencing. Fruit peel samples from different plants were stored in individually labeled cryotubes, and RNA and metabolites were extracted independently for each biological replicate without pooling samples from different plants. RT–qPCR was performed using the same independently processed RNA samples, and transcriptomic and metabolomic data were analyzed at the individual biological-replicate level.

### 4.6. Transcriptome and RT–qPCR Analysis of Mango Peel from Red, Yellow, and Green Cultivars

The experimental samples described above, comprising three biological replicates for each cultivar, were used for transcriptome sequencing and RT–qPCR analysis. Total RNA was extracted from mango fruit peel using the RNAprep Pure Plant Plus Kit (Polysaccharide- and Polyphenolic-rich; Tiangen, China). RNA integrity was assessed by electrophoresis on a 1% agarose gel electrophoresis and spectrophotometry (A260/A280 and A260/A230). Each cultivar contained three biological replicates. Transcriptomic sequencing libraries were constructed from total RNA using a strand-specific mRNA library preparation kit with poly(A) enrichment. Libraries were sequenced on an Illumina platform with a PE150 strategy, generating approximately 6 Gb of clean data per sample. Raw reads were quality-checked with FastQC (v0.11.9) and trimmed using fastp (v0.23.2) [54]. Clean reads were mapped to the reference genome ‘Alphonso’ [36] using HISAT2 (v2.2.1) [55], and gene-level counts were obtained with featureCounts (v2.0.4) [56]. Differential expression analysis between sample groups was performed using DESeq2 (v1.48.2) [57] based on normalized raw read count data. FPKM values were calculated and used to quantify gene expression levels for visualization. *p* values were adjusted for multiple testing using the Benjamini–Hochberg method to obtain the False Discovery Rate (FDR). Genes with an adjusted *p* value (FDR) < 0.05 and |log_2_FC| ≥ 1 were considered significantly differentially expressed. Hierarchical clustering heatmaps were generated in R based on a gene expression matrix derived from transcriptomic data [58]. For RT–qPCR, total RNA was reverse-transcribed into complementary DNA using the HiScript II 1st Strand cDNA Synthesis Kit (+gDNA wiper; Vazyme, Nanjing, China), and qPCR amplification was performed using ChamQ Universal SYBR qPCR Master Mix (Vazyme, Nanjing, China) on a QuantStudio 6 Flex Real-Time PCR System (Thermo Fisher Scientific, Waltham, MA, USA). *MiActin* was selected as an internal reference gene [1]. Amplification efficiencies were estimated using LinRegPCR (2021.2) [59] by linear regression on the log-linear phase of each individual amplification curve. The mean efficiency for each primer pair was calculated from all valid reactions and used to assess primer performance. Relative expression levels were calculated using the 2^−ΔΔCt^ method [60], with the expression level of each *MiR2R3-MYB* gene in ‘GF’ set to 1 and corresponding levels in ‘QP’ and ‘JH’ calculated relative to ‘GF’.

### 4.7. Prediction of MYB-Binding Cis-Elements in Anthocyanin Structural Genes Promoters

To investigate the potential role of differentially expressed anthocyanin structural genes in regulating mango peel coloration, we used the JASPAR 2026 database to predict MYB-binding sites in the promoter regions of anthocyanin structural genes (https://jaspar.elixir.no/; accessed on 18 October 2026) [61]. The search was conducted with the taxonomic group set to Plantae, the CORE collection selected, all species included, and the latest database version used. The MYB family was selected, and the relative profile score threshold was set to 1. The predicted binding sites were merged, removing redundant predicted binding sites.

### 4.8. Analysis of the Anthocyanin Metabolome

The anthocyanin composition and content were determined using the same mango peel samples used for transcriptome analysis, with three biological replicates per cultivar. The fruit peels were lyophilized and finely powdered, and approximately 100 mg of the powder was extracted with 0.6 mL of 70% methanol. After extraction, the samples were incubated overnight at 4 °C, with intermittent vortex mixing performed six times during incubation. The samples were then centrifuged at 10,000× *g* for 10 min, and the supernatants were filtered through 0.22 µm membrane filters and stored until analysis by targeted liquid chromatography–mass spectrometry (LC–MS).

Ultra-performance liquid chromatography–tandem mass spectrometry (UPLC–MS/MS) analysis was performed using an Agilent SB-C18 column (1.8 μm, 2.1 mm × 100 mm) (Agilent Technologies, Santa Clara, CA, USA) maintained at 40 °C. The mobile phase consisted of water containing 0.1% formic acid (solvent A) and acetonitrile (solvent B), delivered at a flow rate of 0.35 mL/min. Gradient elution began with 5% solvent B at 0 min and increased linearly to 95% over 9 min. The proportion of solvent B was held at 95% for 1 min, then decreased to 5% from 10.00 to 11.10 min and maintained at 5% until 14 min to allow for column re-equilibration. A 4 μL aliquot of each sample was injected for analysis. Metabolite annotation was performed by comparing the acquired MS/MS spectra with the reference entries in the Metware database. Quantitative analysis was conducted using a triple–quadrupole mass spectrometer (SCIEX, Framingham, MA, USA) operated in multiple reaction monitoring mode, and the data were processed using Analyst 1.6.3 software. Differential metabolites were identified using OPLS-DA with a fold change cutoff of >2 [62].

### 4.9. Correlation Analysis and Network Construction

Pearson’s correlation analysis was performed using R (v4.5.2) software to assess the relationships between the DEGs and DAMs. Gene–metabolite pairs were considered significantly correlated when the absolute Pearson correlation coefficient (|r|) > 0.8 and the *p*-value < 0.05. Regulatory networks were subsequently constructed for MiR2R3-MYB genes that exhibited significant correlations with DAMs and differential expression patterns among the red (‘GF’), yellow (‘JH’), and green (‘QP’) peel-color groups. The resulting networks were visualized using Cytoscape version 3.8.2 [63].

### 4.10. Prediction and Analysis of Subcellular Localization

The subcellular localization of MiR2R3-MYB proteins was predicted using Plant-mPLoc (http://www.csbio.sjtu.edu.cn/bioinf/plant-multi/; accessed on 16 July 2026) [64]. To experimentally validate this prediction, the coding sequence of MiMYB107 was cloned into the pBWA(V)H2STMVΩ-eGFP vector to generate an MiMYB107–eGFP construct. The recombinant plasmid was transformed into *Agrobacterium tumefaciens* strain GV3101 and transiently expressed in the leaves of 4- to 5-week-old *Nicotiana benthamiana* plants at an optical density (OD_600_) of 0.6. Plants infiltrated with the eGFP vector were used as negative controls. Fluorescence was observed using a Nikon C2-ER confocal laser-scanning microscope, with excitation at 488 nm and emission detection at 510 nm.

### 4.11. Western Blotting Analysis

For Western blotting analysis, leaves from infiltrated *N. benthamiana* plants were collected and immediately ground in liquid nitrogen. Equal amounts of protein samples were separated by sodium dodecyl sulfate–polyacrylamide gel electrophoresis and subsequently transferred onto polyvinylidene fluoride membranes by wet transfer. The membranes were then blocked and incubated overnight with an anti-GFP antibody, followed by incubation with the corresponding horseradish peroxidase-conjugated goat anti-mouse IgG (H + L) secondary antibody for 1 h. A mouse monoclonal antibody specific to plant actin was used to detect actin as a loading control. Chemiluminescent signals were detected using the WesternBright ECL kit and visualized with an automated imaging system.

## 5. Conclusions

In this study, we conducted a genome-wide analysis of the *R2R3-MYB* gene family and identified 194 *MiR2R3-MYB* genes, which were classified into 39 subfamilies based on phylogenetic analysis. Gene structure analysis revealed largely conserved features. Duplication analysis indicated that tandem and segmental duplications were the primary contributors to the expansion of the *MiR2R3-MYB* gene family. Comparative synteny analysis identified more orthologous and collinear *R2R3-MYB* gene pairs between *M. indica* and *P. persica*, indicating a higher level of syntenic conservation within the *R2R3-MYB* gene family between these two species. In addition, 143 putative MYB-binding sites were found in the promoters of anthocyanin DEGs. Integrated transcriptomic and metabolomic analyses of red, yellow, and green mango peels identified 45 DEGs significantly correlated with 36 anthocyanin DAMs. Eight candidate genes identified as central and highly connected nodes in the regulatory network were selected for RT–qPCR validation. *MiMYB107* showed the highest number of positive associations with anthocyanin DEGs. The MiMYB107-eGFP fusion protein was successfully expressed, and its nuclear localization was experimentally confirmed. Its expression was further verified by Western blotting. These findings provide valuable insights into the potential involvement of *MiR2R3-MYB* genes in the regulatory networks underlying anthocyanin biosynthesis in mango.

## Figures and Tables

**Table 1:** Summary of cis-acting elements in the promoters of *MiR2R3-MYB* genes.

Category	Function	Cis-Elements	Number
light responsive	light responsive	Box4, G-box, GT1-motif, TCT-motif, GATA-motif, and others	2573
stress responsive	anaerobic induction	ARE	369
	anoxic specific inducibility data	GC-motif	8
	defense and stress	TC-rich repeats	91
	drought-inducibility	MBS	121
	low-temperature responsiveness	LTR	83
	maximal elicitor-mediated activation	AT-rich sequence	16
	wound-responsive	WUN-motif	9
plant growth	cell cycle regulation	MSA-like	7
	differentiation of the palisade mesophyll cells	HD-Zip 1	19
	meristem expression	CAT-box	82
	seed-specific regulation	RY-element	23
	zein metabolism regulation	O2-site	95
	circadian control	circadian	54
Phytohormone responsive	abscisic acid	ABRE	385
	auxin-responsive	TGA-element, AuxRR-core	123
	endosperm regulation	GCN4_motif	65
	flavonoid biosynthetic	MBSI	5
	gibberellin-responsiveness	P-box, TATC-box, GARE-motif	163
	MeJA-responsiveness	CGTCA-motif, TGACG-motif	454
	salicylic acid responsiveness	TCA-element, SARE	105
Total			4850

## Data Availability

The data that support the findings of this study are available from the corresponding authors upon reasonable request due to privacy.

## Supplementary Materials

The following supporting information can be downloaded at https://www.mdpi.com/article/10.3390/plants15213427/s1. Table S1: Protein sequence information for 370 *MiMYB* genes; Table S2: Characteristic features of 194 MiR2R3-MYB proteins; Table S3: Tandem Genes of *MiR2R3-MYB* gene pairs in *M. indica*; Table S4: 125 AtR2R3-MYB protein sequences; Table S5: Conserved motif composition of MiR2R3-MYB proteins in *M. indica;* Table S6: Cis-regulatory elements in *MiR2R3-MYB* promoter regions; Table S7: Intragenomic collinearity pairs among *MiR2R3-MYB* genes in *M. indica;* Table S8: Information on duplicated *MiR2R3-MYB* gene pairs on chromosome 3 in *M. indica;* Table S9: Orthologous relationships of *M. indica R2R3-MYB* genes with *A. thaliana*, *M. acuminata*, and *P.s persica*; Table S10: RNA-seq result; Table S11: Comparison results of sequencing reads and reference genome; Table S12: FPKM values of differentially expressed *MiR2R3-MYB* genes; Table S13: FPKM values of anthocyanin structural genes; Table S14: Prediction of MYB-binding cis-elements in the promoters of anthocyanin structural genes; Table S15: DAMs appearing specifically at different group; Table S16: The correlation coefficients between *MiR2R3-MYB* DEGs and anthocyanin DAMs; Table S17: List of RT-qPCR primer sequences; Figure S1: Analysis of cis-regulatory elements in *MiR2R3-MYB* gene promoters; Figure S2: Principal component analysis (PCA) of peel samples from three mango cultivars (‘GF’, ‘JH’, and ‘QP’). Each point represents an independent biological replicate. Ellipses indicate the 95% confidence interval. PC1 and PC2 explain 35.09% and 30.20% of the total variance, respectively; Figure S3: Pearson correlation heatmap of *MiR2R3-MYB* DEGs and anthocyanin DAMs. Red and green represent positive and negative correlations, respectively. Asterisks indicate statistically significant correlations (* *p* < 0.05, ** *p* < 0.01, and *** *p* < 0.001); Figure S4: Integrity verification of total RNA isolated from ‘GF’, ‘JH’, and ‘QP’ mango peels. Samples were separated on a 1.0% agarose gel. The sharp 28S and 18S ribosomal subunit bands demonstrate that the RNA was of high quality and suitable for subsequent transcriptome sequencing. M: DNA Marker; Figure S5: RT-qPCR melting curves for the eight candidate *MiR2R3-MYB* genes and the *MiActin* reference gene; Figure S6: Subcellular localization of the MiMYB107-eGFP fusion protein in epidermal cells of *N. benthamiana* leaves. Free eGFP was used as a control and imaged under identical confocal settings. GFP, green fluorescent protein; Chloroplast, chloroplast auto-fluorescence; Bright, bright field; Merge, merge field. Scale bars = 20 μm. MiMYB107-eGFP(a), MiMYB107-eGFP(b), MiMYB107-eGFP(c) represent three replicates (*n* = 3).

## Abbreviations

The following abbreviations are used in this manuscript:

TFs	Transcription factors
MYB TFs	MYB transcription factors
R2R3-MYB TFs	R2R3-MYB transcription factors
CDS	Coding sequence
UTR	Untranslated Region
DEGs	Differentially expressed genes
FPKM	Fragments per kilobase of transcript per million mapped reads
RT-qPCR	Reverse transcription quantitative real-time polymerase chain reaction
LC–MS	Liquid chromatography-mass spectrometry
UPLC–MS/MS	Ultra-performance liquid chromatography-tandem mass spectrometry
DAMs	differentially accumulated metabolites
OPLS-DA	Orthogonal partial least-squares discriminant analysis
FDR	False Discovery Rate
*Ka/Ks*	ratio of nonsynonymous to synonymous substitutions
