# Supplementary material for: Genome-Wide Identification of the R2R3-MYB Gene Family in Mango and Multi-Omics Analyses Reveal Genes Involved in Anthocyanin Biosynthesis in Fruit Peels of Different Colors
Source: Plants (Basel). 2026 Nov 4;15(21):3427. doi: 10.3390/plants15213427 (PMC13765629; doi:10.3390/plants15213427)
Supplement: Supplementary file 1 [file plants-15-03427-s001.zip › Figure S1.pdf]

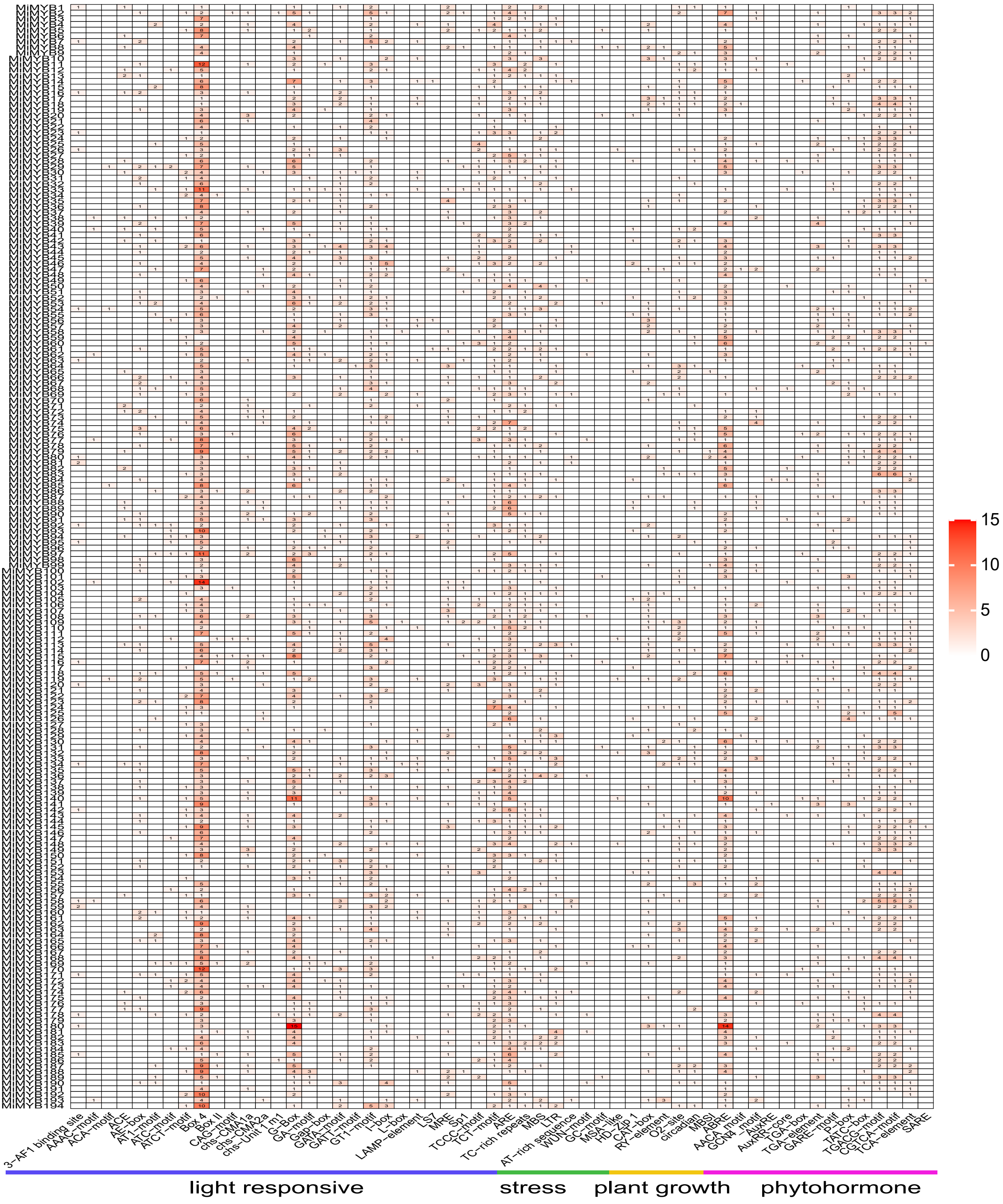
3-AF1 binding site
AAAC-motif
ACA-motif
ACE
AE-box
AT1-motif
ATC-motif
ATCT-motif
Box 4
Box II
CAG-motif
chs-CMA1a
chs-CMA2a
chs-Unit 1 m1
G-Box
GA-motif
Gap-box
GATA-motif
GATT-motif
GT1-motif
I-box
L-box
LAMP-element
LS7
MRE
Sp1
TCCC-motif
TCT-motif
ARE
TC-rich repeats
MBS
LTR
AT-rich sequence
WUN-motif
GC-motif
MSA-like
HD-Zip 1
CAT-box
RY-element
O2-site
circadian
MBSI
ABRE
AACA_motif
GCN4_motif
AuxRE
AuxRR-core
TGA-box
TGA-element
GARE-motif
P-box
TATC-box
TGACG-motif
CGTCA-motif
TCA-element
SARE
light responsive
stress
plant growth
phytohormone
15
10
5
0
