# Supplementary material for: Genome-Wide Identification of the R2R3-MYB Gene Family in Mango and Multi-Omics Analyses Reveal Genes Involved in Anthocyanin Biosynthesis in Fruit Peels of Different Colors
Source: Plants (Basel). 2026 Nov 4;15(21):3427. doi: 10.3390/plants15213427 (PMC13765629; doi:10.3390/plants15213427)
Supplement: Supplementary file 1 [file plants-15-03427-s001.zip › Figure S2.pdf]

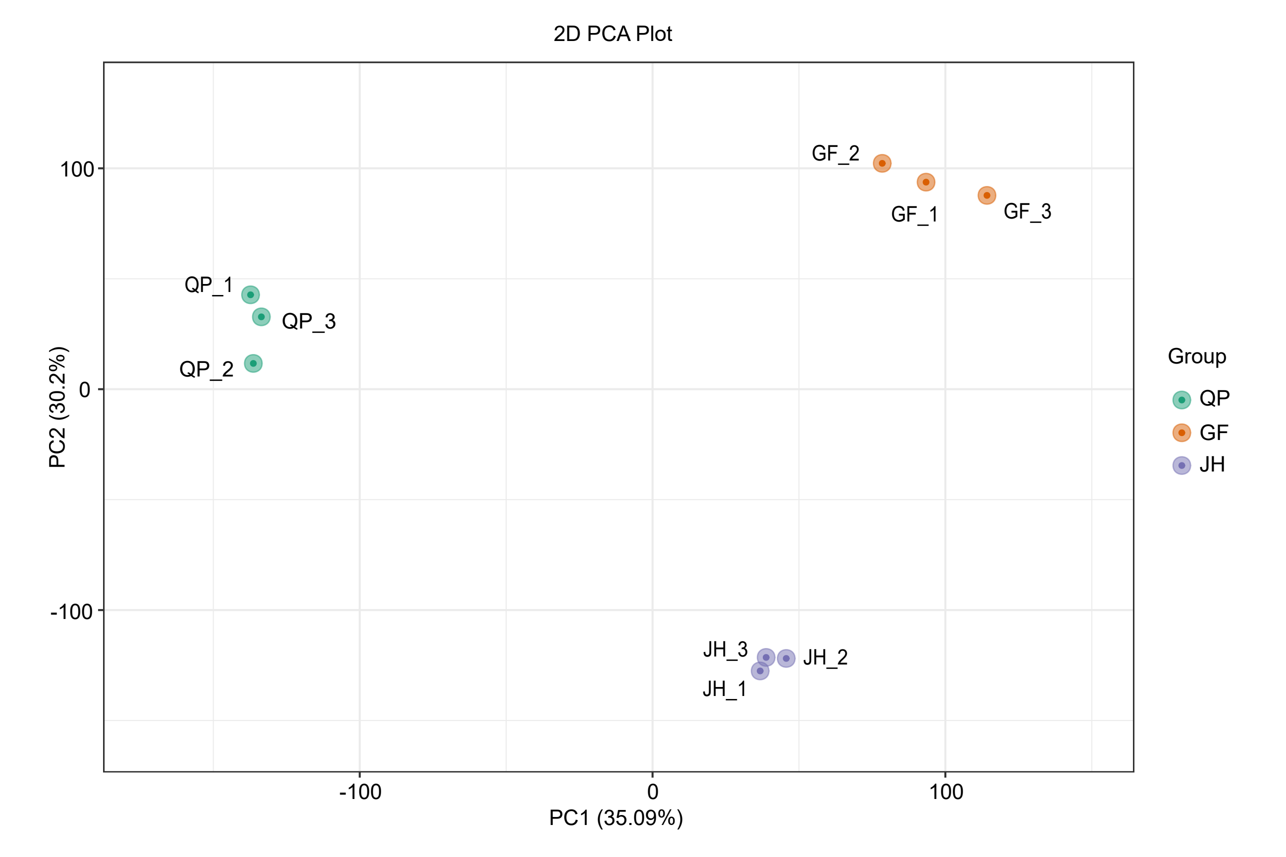
2D PCA Plot
GF_2
GF_1
GF_3
QP_1
QP_3
QP_2
JH_3
JH_2
JH_1
100
0
-100
-100
0
100
PC1 (35.09%)
PC2 (30.2%)
Group
QP
GF
JH
