# Supplementary material for: Genome-Wide Identification of the R2R3-MYB Gene Family in Mango and Multi-Omics Analyses Reveal Genes Involved in Anthocyanin Biosynthesis in Fruit Peels of Different Colors
Source: Plants (Basel). 2026 Nov 4;15(21):3427. doi: 10.3390/plants15213427 (PMC13765629; doi:10.3390/plants15213427)
Supplement: Supplementary file 1 [file plants-15-03427-s001.zip › Figure S3.pdf]

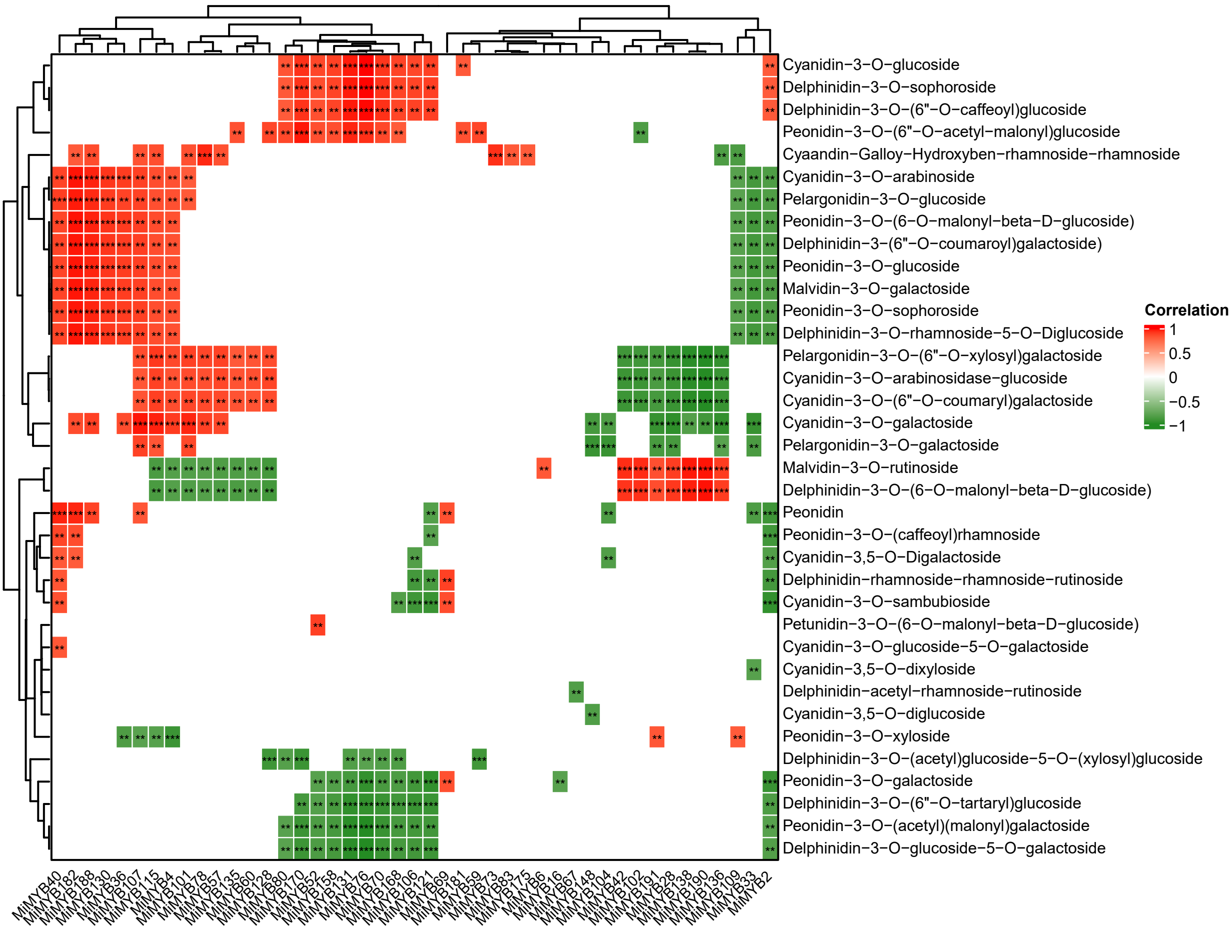
Cyanidin−3−O−glucoside
Delphinidin−3−O−sophoroside
Delphinidin−3−O−(6"−O−caffeoyl)glucoside
Peonidin−3−O−(6"−O−acetyl−malonyl)glucoside
Cyaandin−Galloy−Hydroxyben−rhamnoside−rhamnoside
Cyanidin−3−O−arabinoside
Pelargonidin−3−O−glucoside
Peonidin−3−O−(6−O−malonyl−beta−D−glucoside)
Delphinidin−3−(6"−O−coumaroyl)galactoside)
Peonidin−3−O−glucoside
Malvidin−3−O−galactoside
Peonidin−3−O−sophoroside
Delphinidin−3−O−rhamnoside−5−O−Diglucoside
Pelargonidin−3−O−(6"−O−xylosyl)galactoside
Cyanidin−3−O−arabinosidase−glucoside
Cyanidin−3−O−(6"−O−coumaryl)galactoside
Cyanidin−3−O−galactoside
Pelargonidin−3−O−galactoside
Malvidin−3−O−rutinoside
Delphinidin−3−O−(6−O−malonyl−beta−D−glucoside)
Peonidin
Peonidin−3−O−(caffeoyl)rhamnoside
Cyanidin−3,5−O−Digalactoside
Delphinidin−rhamnoside−rhamnoside−rutinoside
Cyanidin−3−O−sambubioside
Petunidin−3−O−(6−O−malonyl−beta−D−glucoside)
Cyanidin−3−O−glucoside−5−O−galactoside
Cyanidin−3,5−O−dixyloside
Delphinidin−acetyl−rhamnoside−rutinoside
Cyanidin−3,5−O−diglucoside
Peonidin−3−O−xyloside
Delphinidin−3−O−(acetyl)glucoside−5−O−(xylosyl)glucoside
Peonidin−3−O−galactoside
Delphinidin−3−O−(6"−O−tartaryl)glucoside
Peonidin−3−O−(acetyl)(malonyl)galactoside
Delphinidin−3−O−glucoside−5−O−galactoside
Correlation
1
0.5
0
−0.5
−1
MiMYB40
MiMYB182
MiMYB188
MiMYB130
MiMYB36
MiMYB107
MiMYB115
MiMYB4
MiMYB101
MiMYB78
MiMYB57
MiMYB135
MiMYB60
MiMYB128
MiMYB80
MiMYB170
MiMYB52
MiMYB158
MiMYB131
MiMYB76
MiMYB70
MiMYB168
MiMYB106
MiMYB121
MiMYB69
MiMYB181
MiMYB59
MiMYB73
MiMYB83
MiMYB175
MiMYB6
MiMYB16
MiMYB67
MiMYB148
MiMYB104
MiMYB42
MiMYB102
MiMYB191
MiMYB28
MiMYB138
MiMYB190
MiMYB136
MiMYB109
MiMYB33
MiMYB2
