# Supplementary material for: Genome-Wide Identification of the R2R3-MYB Gene Family in Mango and Multi-Omics Analyses Reveal Genes Involved in Anthocyanin Biosynthesis in Fruit Peels of Different Colors
Source: Plants (Basel). 2026 Nov 4;15(21):3427. doi: 10.3390/plants15213427 (PMC13765629; doi:10.3390/plants15213427)
Supplement: Supplementary file 1 [file plants-15-03427-s001.zip › Figure S4.pdf]

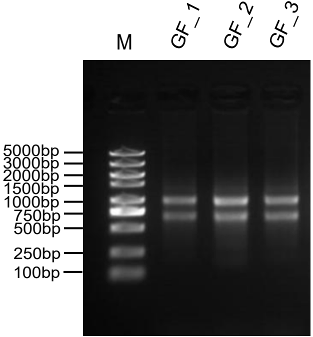
M
GF_1
GF_2
GF_3
5000bp
3000bp
2000bp
1500bp
1000bp
750bp
500bp
250bp
100bp

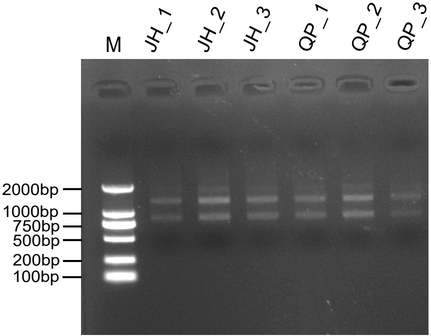
M
JH_1
JH_2
JH_3
QP_1
QP_2
QP_3
2000bp
1000bp
750bp
500bp
200bp
100bp
