# Supplementary material for: Genome-Wide Identification of the R2R3-MYB Gene Family in Mango and Multi-Omics Analyses Reveal Genes Involved in Anthocyanin Biosynthesis in Fruit Peels of Different Colors
Source: Plants (Basel). 2026 Nov 4;15(21):3427. doi: 10.3390/plants15213427 (PMC13765629; doi:10.3390/plants15213427)
Supplement: Supplementary file 1 [file plants-15-03427-s001.zip › FigureS6.pdf]

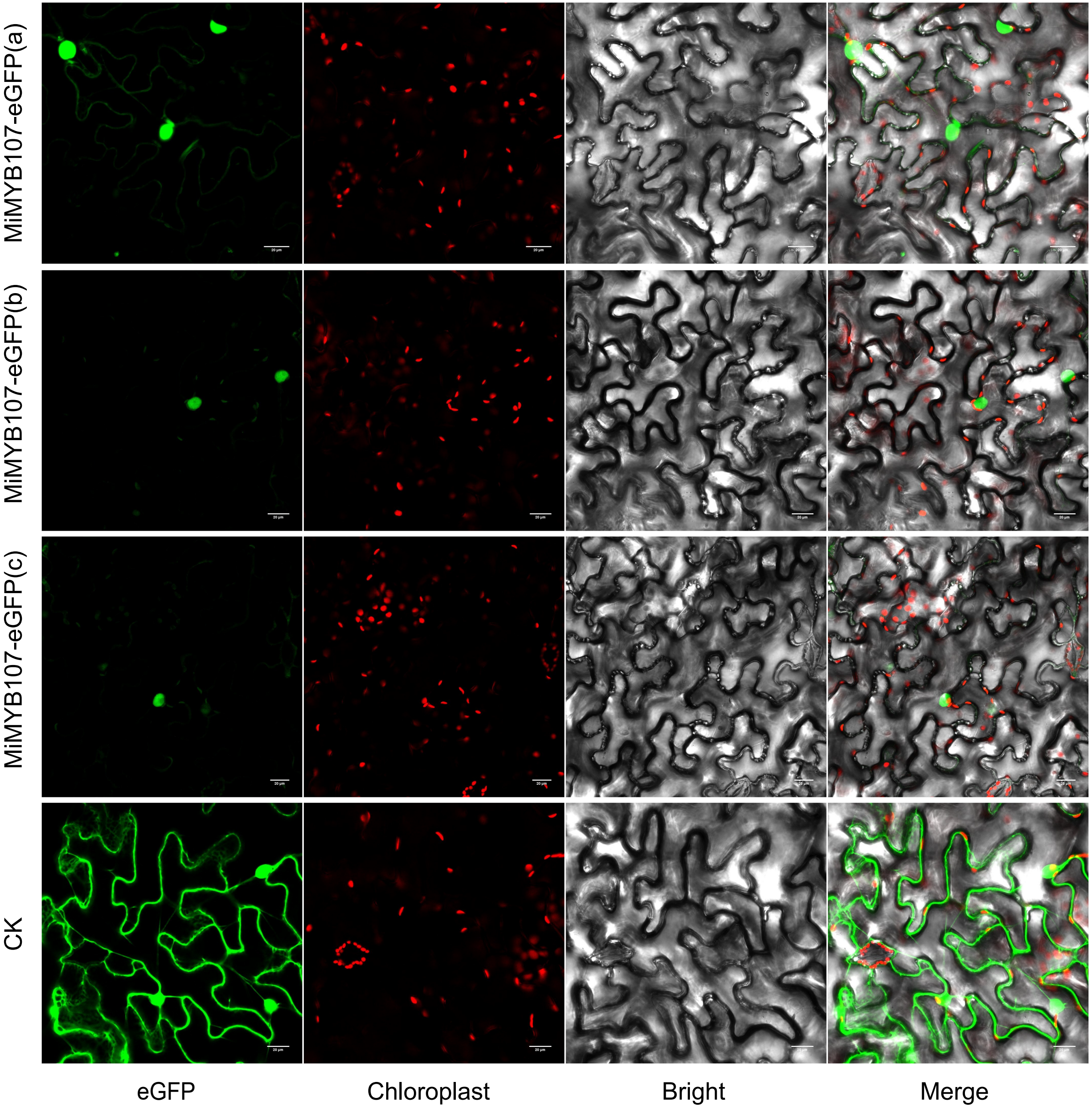

MiMYB107-eGFP(a)
MiMYB107-eGFP(b)
MiMYB107-eGFP(c)
CK
eGFP
Chloroplast
Bright
Merge
20 μm

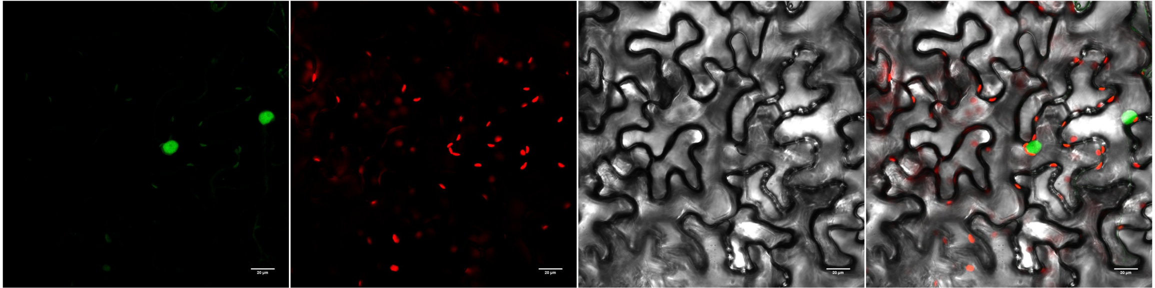
20 μm
20 μm
20 μm
20 μm

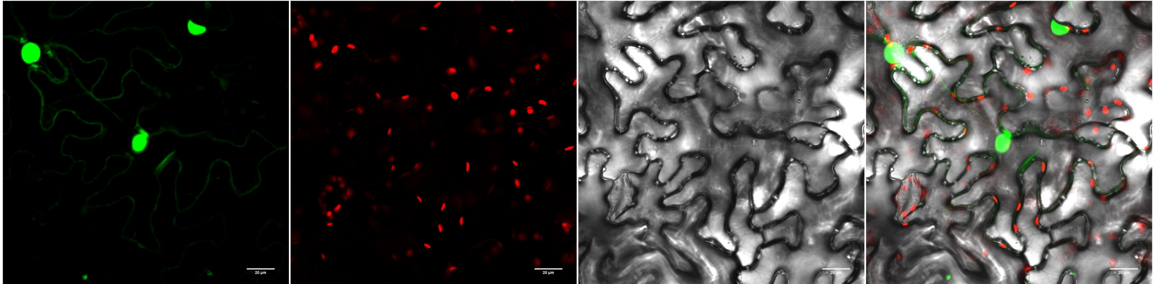
20 µm
20 µm
20 µm
20 µm
