# Supplementary material for: Genome-Wide Identification of the R2R3-MYB Gene Family in Mango and Multi-Omics Analyses Reveal Genes Involved in Anthocyanin Biosynthesis in Fruit Peels of Different Colors
Source: Plants (Basel). 2026 Nov 4;15(21):3427. doi: 10.3390/plants15213427 (PMC13765629; doi:10.3390/plants15213427)
Supplement: Supplementary file 1 [file plants-15-03427-s001.zip › Supplementary Figure.pdf]

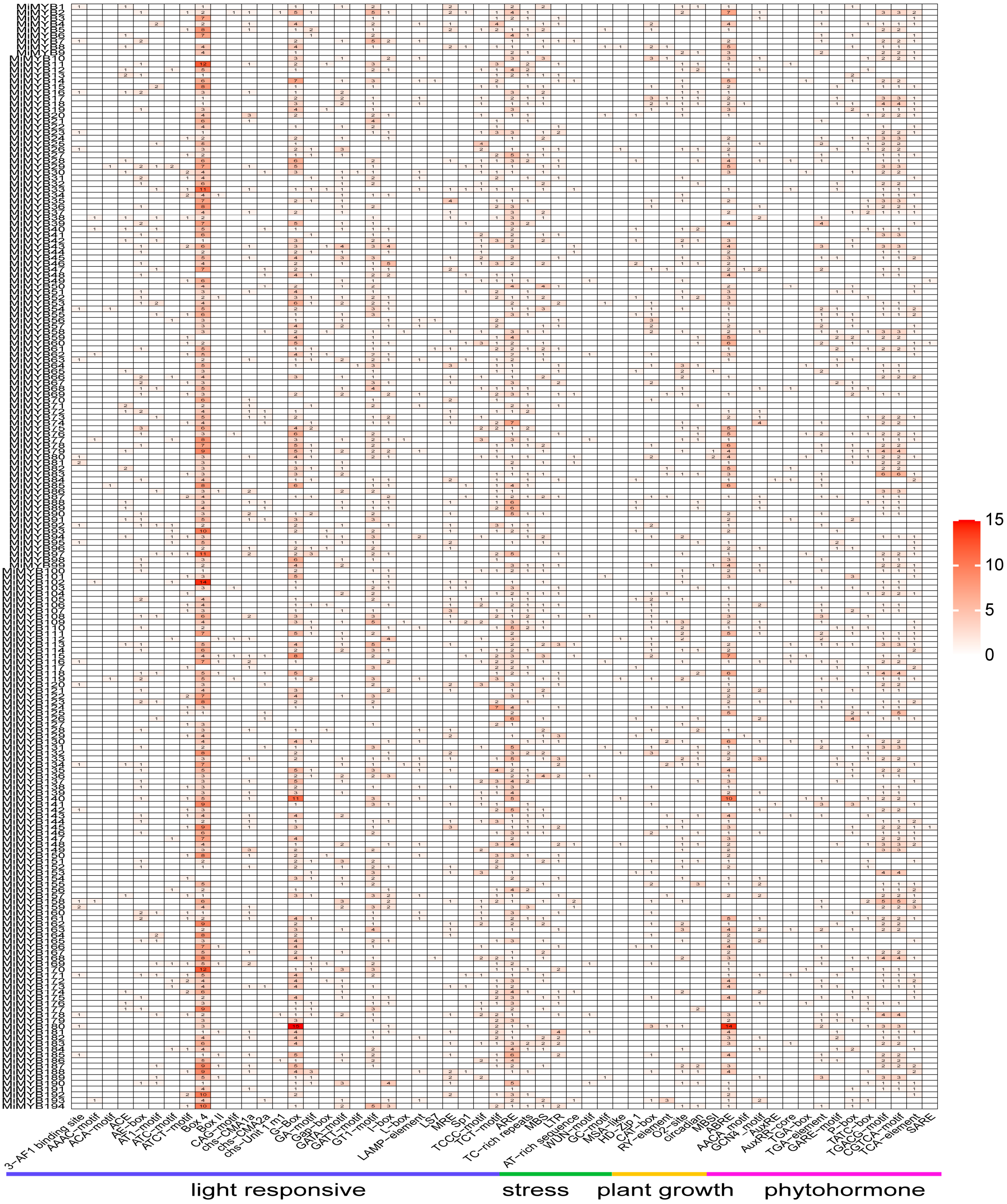

Figure S1. Analysis of cis-regulatory elements in *MiR2R3-MYB* gene promoters.

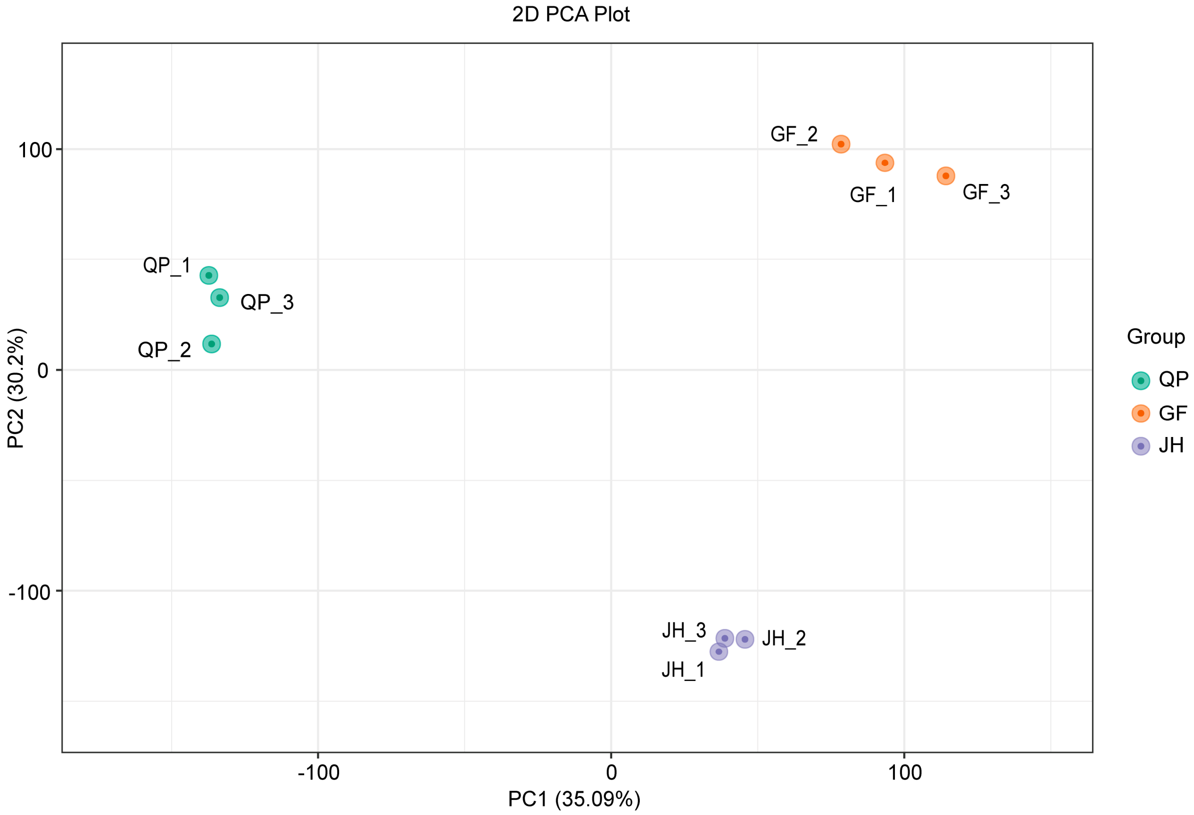

Figure S2. Principal component analysis (PCA) of peel samples from three mango cultivars ('GF', 'JH', and 'QP'). Each point represents an independent biological replicate. Ellipses indicate the 95% confidence interval. PC1 and PC2 explain 35.09% and 30.20% of the total variance, respectively.

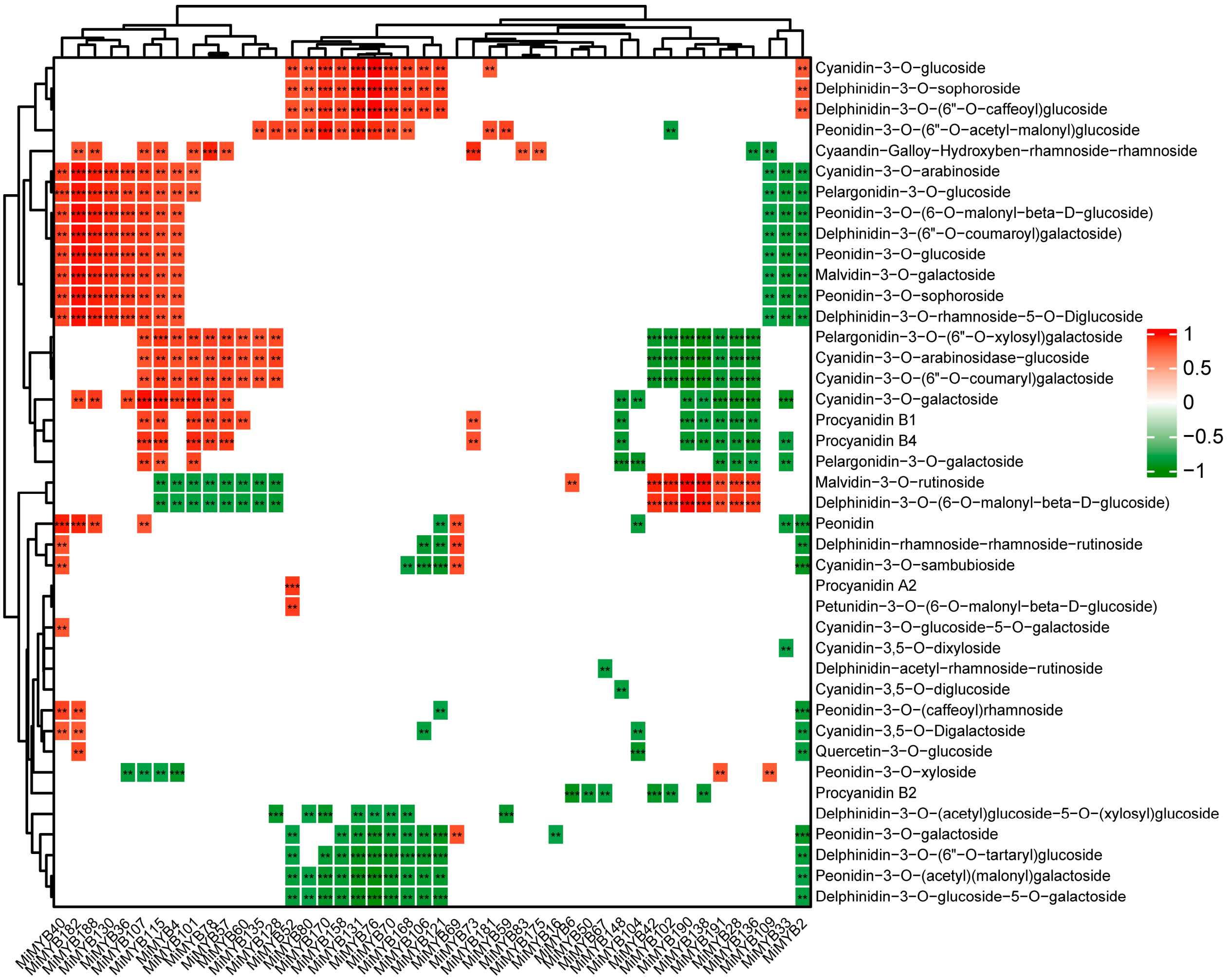

Figure S3. Pearson correlation heatmap of MiR2R3-MYB DEGs and anthocyanin DAMs. Red and green representing positive and negative correlations, respectively. Asterisks indicate statistically significant correlations (*p < 0.05, **p < 0.01, and ***p < 0.001).

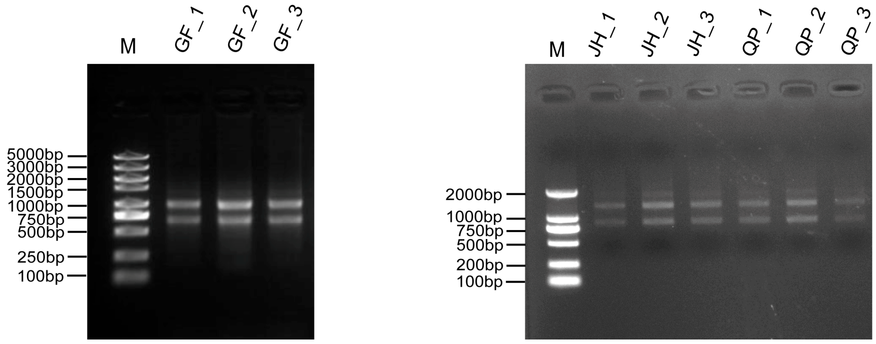

Figure S4. Integrity verification of total RNA isolated from 'GF', 'JH', and 'QP' mango peels. Samples were separated on a 1.0% agarose gel. The sharp 28S and 18S ribosomal subunit bands demonstrate that the RNA was of high quality and suitable for subsequent transcriptome sequencing. M: DNA Marker.

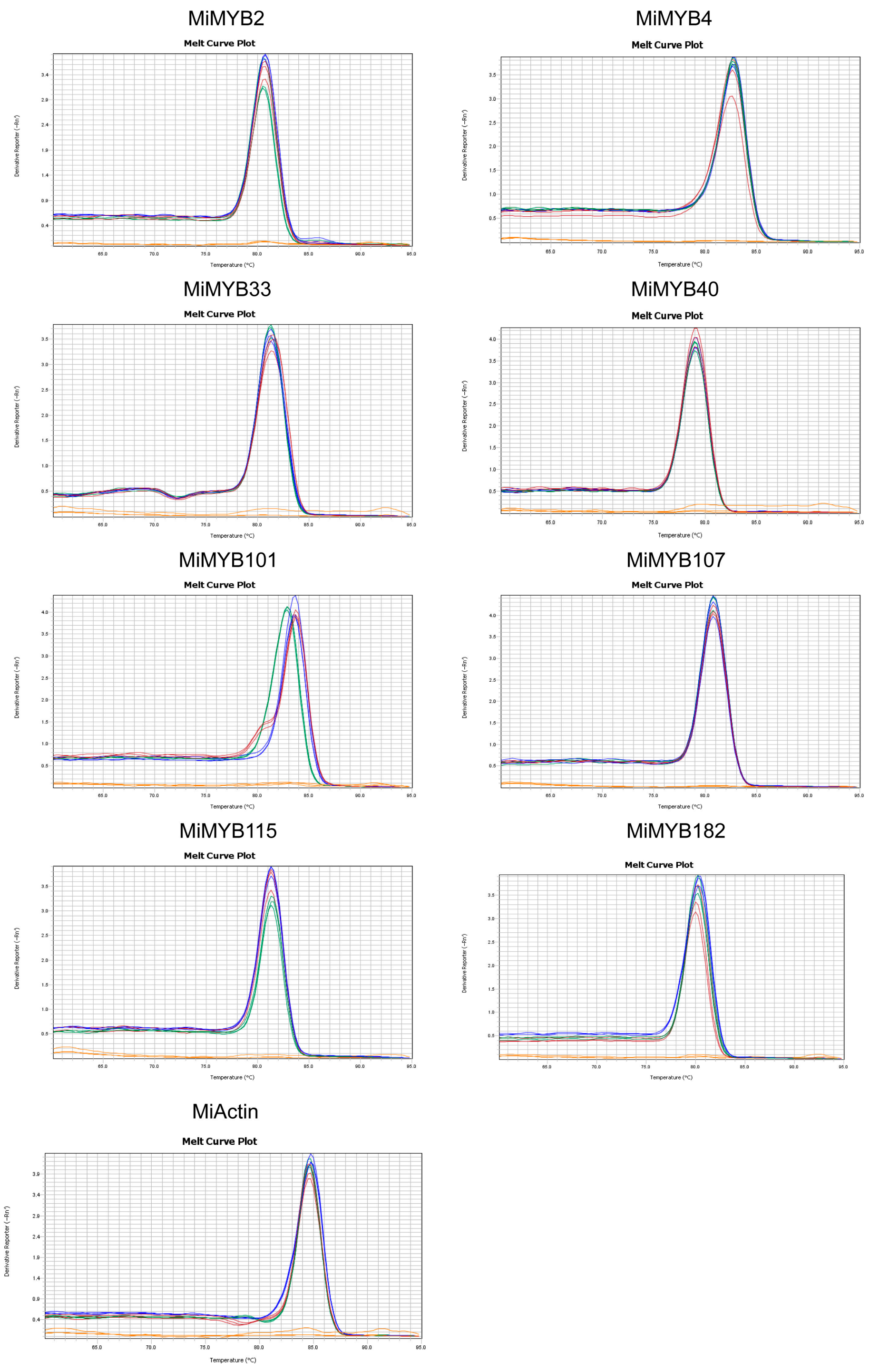

Figure S5. RT-qPCR melting curves for the eight candidate *MiR2R3-MYB* genes and the *MiActin* reference gene.

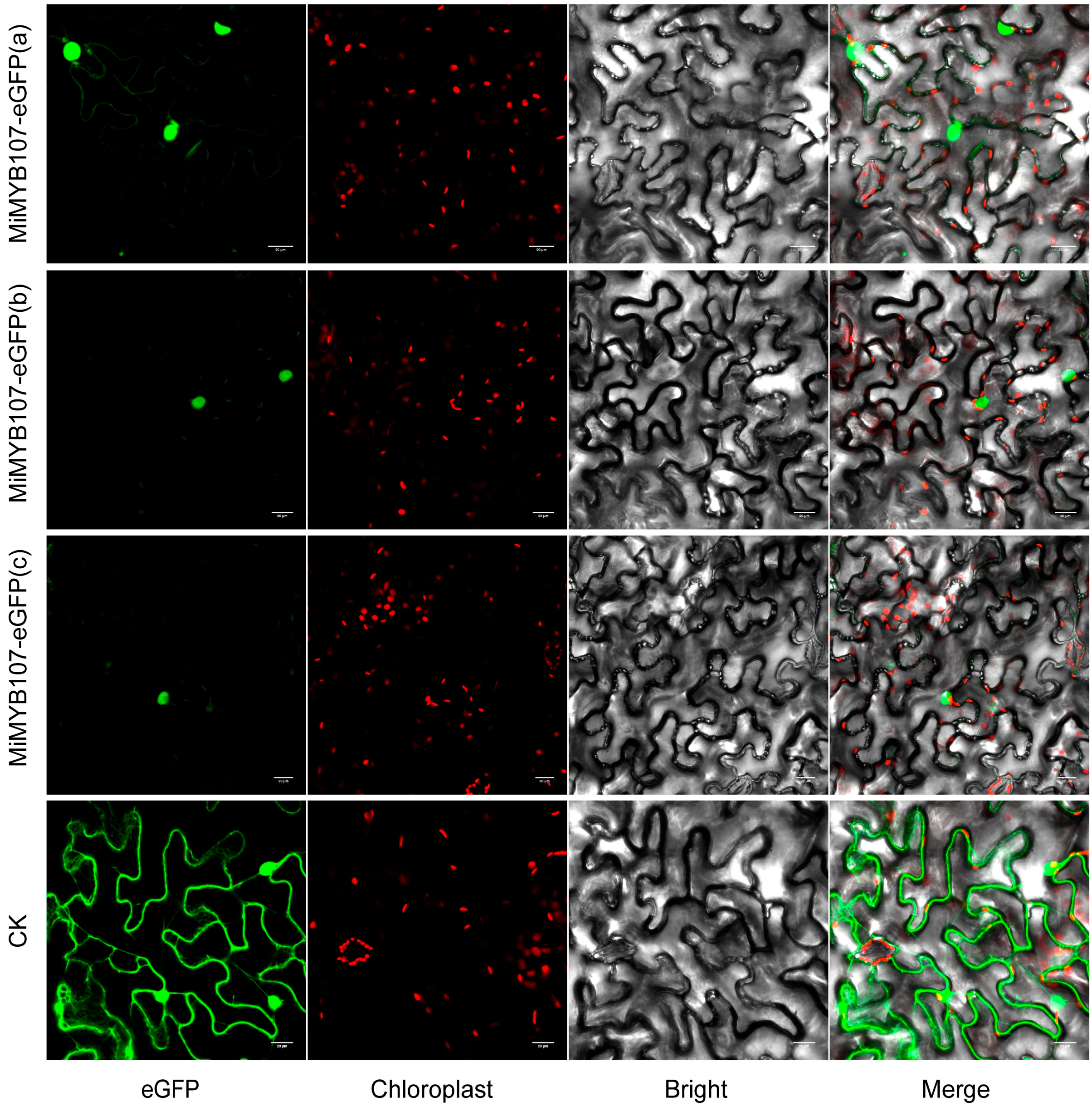

Figure S6. Subcellular localization of the MiMYB107-eGFP fusion protein in epidermal cells of N. benthamiana leaves. Free eGFP was used as a control and imaged under identical confocal settings. GFP, green fluorescent protein; Chloroplast, chloroplast autofluorescence; Bright, bright field; Merge, merge field. Scale bars = 20 μm. MiMYB107-eGFP(a), MiMYB107-eGFP(b), MiMYB107-eGFP(c) represent three replicates (n = 3).
